# Effectiveness of a Lifestyle Intervention for People With a Severe Mental Illness in Dutch Outpatient Mental Health Care: A Randomized Clinical Trial

**DOI:** 10.1001/jamapsychiatry.2023.1566

**Published:** 2023-06-21

**Authors:** Florine Sanna Walburg, Berno van Meijel, Trynke Hoekstra, Jelle Kol, Laura Michelle Pape, Johanna Willemina de Joode, Maurits van Tulder, Marcel Adriaanse

**Affiliations:** 1 Department Health Sciences, Vrije Universiteit Amsterdam, Amsterdam Public Health Research Institute, Amsterdam, the Netherlands; 2 Inholland University of Applied Sciences, Department of Health, Sports and Welfare, Mental Health Nursing Research Group, Amsterdam, the Netherlands; 3 Department of Psychiatry, Amsterdam Public Health Research Institute, Amsterdam UMC (VUmc), Amsterdam, the Netherlands; 4 Parnassia Psychiatric Institute, Parnassia Academy, The Hague, the Netherlands; 5 Department of Clinical, Neuro- and Developmental Psychology, Faculty of Behavioral and Movement Sciences, Vrije Universiteit Amsterdam, Amsterdam, the Netherlands

## Abstract

**Importance:**

People with a severe mental illness (SMI) have a life expectancy reduced by 10 to 20 years compared with the general population, primarily attributable to cardiometabolic disorders. Lifestyle interventions for people with SMI can improve health and reduce cardiometabolic risk.

**Objective:**

To evaluate the effectiveness of a group-based lifestyle intervention among people with SMI in outpatient treatment settings compared with treatment as usual (TAU).

**Design, Setting, and Participants:**

The Severe Mental Illness Lifestyle Evaluation (SMILE) study is a pragmatic cluster randomized clinical trial performed in 8 mental health care centers with 21 flexible assertive community treatment teams in the Netherlands. Inclusion criteria were SMI, age of 18 years or older, and body mass index (calculated as weight in kilograms divided by height in meters squared) of 27 or greater. Data were collected from January 2018 to February 2020, and data were analyzed from September 2020 to February 2023.

**Interventions:**

Weekly 2-hour group sessions for 6 months followed by monthly 2-hour group sessions for another 6 months, delivered by trained mental health care workers. The intervention targeted overall lifestyle changes, emphasizing establishing a healthy diet and promoting physical activity. TAU (control) did not include structured interventions or advice on lifestyle.

**Main Outcomes and Measures:**

Crude and adjusted linear mixed models and multivariable logistic regression analyses were performed. The main outcome was body weight change. Secondary outcomes included changes in body mass index, blood pressure, lipid profiles, fasting glucose level, quality of life, self-management ability, and lifestyle behaviors (physical activity and health, mental health, nutrition, and sleep).

**Results:**

The study population included 11 lifestyle intervention teams (126 participants) and 10 TAU teams (98 participants). Of 224 included patients, 137 (61.2%) were female, and the mean (SD) age was 47.6 (11.1) years. From baseline to 12 months, participants in the lifestyle intervention group lost 3.3 kg (95% CI, −6.2 to −0.4) more than those in the control group. In the lifestyle intervention group, people with high attendance rates lost more weight than participants with medium and low rates (mean [SD] weight loss: high, −4.9 [8.1] kg; medium, −0.2 [7.8] kg; low, 0.8 [8.3] kg). Only small or no changes were found for secondary outcomes.

**Conclusions and Relevance:**

In this trial, the lifestyle intervention significantly reduced weight from baseline to 12 months in overweight and obese adults with SMI. Tailoring lifestyle interventions and increasing attendance rates might be beneficial for people with SMI.

**Trial Registration:**

Netherlands Trial Register Identifier: NTR6837

## Key Points

**Question:**

What is the effectiveness of lifestyle intervention among people with severe mental illness compared with treatment as usual?

**Findings:**

In this pragmatic randomized clinical trial including 224 participants, lifestyle intervention in an outpatient treatment setting demonstrated a significant body weight change (−3.3 kg) compared with treatment as usual from baseline to 12 months.

**Meaning:**

The lifestyle intervention was effective in reducing weight in people with severe mental illness and may lead to reduced risk of cardiometabolic disorders.

## Teaser

This randomized clinical trial evaluates the effectiveness of a group-based lifestyle intervention among people with severe mental illness in outpatient treatment settings compared with treatment as usual.

## Introduction

Morbidity and mortality rates of people with severe mental illness (SMI) are significantly higher than in the general population, with an estimated life expectancy reduction of 10 to 20 years,^1,2,3,4^ primarily attributable to cardiometabolic disorders.^5,6,7,8^ Several factors contribute to this increased risk, including adverse effects of antipsychotic medication, insufficient treatment of somatic diseases, genetic vulnerability, poor access to health care, and unhealthy lifestyles.^9,10,11,12,13^

People with SMI engage less in physical activity, are more sedentary, have a higher calorific intake with excessive fats and sugars, and are more likely to smoke than the general population.^14,15,16,17^ People with SMI could benefit from more healthy lifestyles leading to a better physical and mental health and improved quality of life.^12^ There are multiple lifestyle interventions aiming to assist people with SMI in improving health and reducing cardiometabolic risk.^14,18^ Although some interventions are effective and recommended in clinical guidelines,^12^ cardiometabolic risk factors often remain untreated in people with SMI. Besides, lifestyle interventions are not, or only poorly, implemented in daily practice, and innovative and feasible approaches for effectively delivering these are urgently needed.

We evaluated the effectiveness of a modified version of the Strategies to Reduce Injuries and Develop confidence in Elders (STRIDE) intervention, a group-based lifestyle intervention for people with SMI, in an outpatient treatment setting compared with treatment as usual (TAU). The intervention is based on the STRIDE weight loss and lifestyle intervention for individuals taking antipsychotic medications.^15,16^

## Methods

### Research Design

A pragmatic cluster randomized clinical trial with 1-year follow-up was performed to test the effectiveness of the lifestyle intervention compared with TAU, with recruitment from January 2018 to January 2019. A total of 21 flexible assertive community treatment (FACT) teams from 8 mental health care centers in the Netherlands participated in the trial. Ethical approval was from the Medical Ethical Committee of the VU University Medical Center in Amsterdam. The Severe Mental Illness Lifestyle Evaluation (SMILE) study protocol has been previously published^16^ and can be found in Supplement 1. All participants provided written informed consent. This study followed the Consolidated Standards of Reporting Trials (CONSORT) reporting guideline.

### Setting and Participants

FACT teams are the most common Dutch outpatient mental health care service for long-term care for community-dwelling people with SMI.^17^ A Dutch FACT team is an extended version of an assertive community treatment (ACT) model. Like ACT, FACT teams are outreaching and multidisciplinary, including a psychiatrist, clinical nurse specialist, a psychologist, a mental health nurse who functions as case manager, expert-by-experience, and a supported employment specialist. Also similar to ACT, FACT care includes illness management, symptom treatment, guidance and practical assistance in daily living, rehabilitation, and recovery support. FACT teams differ from ACT teams primarily in that the former are designed to serve the broad range of people with SMIs whereas the latter are provided mainly to a subgroup of similar persons with a history of high acute care service utilization or housing instability. FACT teams have treatment responsibility for all clients with SMI in a specific region, each covering 200 to 250 outpatients. FACT teams offer 2 levels of care: individual case management for most clients and full ACT when there is a need for shared caseload and assertive outreach. In the Netherlands, there are approximately 250 certified FACT teams. FACT teams joined the study on a voluntary basis. FACT teams were approached by research staff with information regarding the study procedures and intervention and were invited to join the study. Through snowball sampling, subsequent teams were approached and asked to join the study.

Eligibility criteria for participants were age 18 years or older, active FACT team care, and body mass index (BMI; calculated as weight in kilograms divided by height in meters squared) of 27 or more (chosen to include those with a higher risk of cardiometabolic disorders, as in the STRIDE study^19^). Exclusion criteria included experiencing cognitive impairment that could interfere with active participation, contraindications for participation (eg, acute psychiatric crisis or stroke), inability to communicate in Dutch, and pregnant, breastfeeding, or planning pregnancy. Nationality (Dutch or non-Dutch) was collected by self-report. Clients of a FACT team that satisfied the inclusion and exclusion criteria, agreed to participate, and provided informed consent participated in the study.

### Randomization and Blinding

Cluster randomization was performed at the level of FACT teams. A total of 22 FACT teams were randomized to the lifestyle intervention or TAU (11 teams in each condition), and 1 FACT team assigned to TAU withdrew their participation in the study. Randomization was performed by an uninvolved statistician using a Microsoft Excel random number function. The nature of the intervention precluded blinding of FACT team staff and participants.

### Lifestyle Intervention

The SMILE intervention is primarily modeled after the successful STRIDE intervention.^19^ The STRIDE lifestyle intervention was developed for persons with SMI. In turn, STRIDE was based on prior research, ie, the PREMIER clinical trial,^20^ behavior change theories, such as the transtheoretical model,^21,22^ and motivational theory.^23,24,25^

In the SMILE study, the session content of the STRIDE intervention program materials was used (eMethods in Supplement 2).^26^ The lifestyle intervention was adapted to fit Dutch food standards and customs. Participants wanting to stop smoking were offered referral for external support, for example, by general practitioners. The lifestyle intervention was carried out by 2 trained mental health workers who were members of that team. The lifestyle intervention’s duration was 12 months and consisted of (1) the initial intervention, with 24 sessions of weekly 2-hour group meetings delivered over the first 6 months, and (2) the maintenance phase, which included 6 monthly group sessions (sessions 25 to 30) focusing on maintaining weight loss. Maintenance sessions were, if needed, supplemented with monthly individual telephone sessions (about 15 minutes) with mental health workers. More information regarding the lifestyle intervention can be found elsewhere.^16,27,28^

### TAU

FACT teams in the control group provided TAU without structured lifestyle interventions or advice on lifestyle changes.

### Assessments

Assessments were performed at baseline and at 6 and 12 months. Body weight was also measured after 3 months to detect short-term changes. Laboratory assessments were only performed at baseline and 12 months. Participants received €30 (US $32.9) compensation for their participation in the study at baseline and at 6 and 12 months in €10 (US $11.0) vouchers at each time point. Assessments were conducted by trained FACT team staff. Research staff helped to conduct assessments when there was a shortage of FACT staff.

### Outcomes

The primary outcome was body weight change; this was measured twice to the nearest 0.1 kg at each time point using a digital scale (Beurer GS210 glass scale; Beurer), with participants wearing only light clothing, and averaged. Changes in BMI were computed as well. Additionally, we report on the percentage of participants who achieved 5% and 10% weight loss from baseline.

Secondary clinical outcomes were systolic and diastolic blood pressures, lipid profiles (total cholesterol, high-density lipoprotein cholesterol, low-density lipoprotein cholesterol, and triglyceride levels), and fasting glucose levels. We could not analyze waist circumference changes because the measurement protocol was not used consistently. Patient-reported secondary outcomes included physical health; mental health; healthy physical activity pattern; healthy nutrition pattern; satisfaction with weight, nutrition, physical activity, and sleep in the previous 2 weeks (all on a linear numeric scale from 0 to 10); mean hours of night sleep (previous 2 weeks); quality of life (12-Item Short Form Survey [SF-12])^29^; and self-management (Patient’s Activation Measure [PAM-13]).^30^ The SF-12, a subjective report of the individual’s physical and mental health functioning, yielded 2 subscales: the Physical Component Summary and the Mental Component Summary. Scores range from 0 to 100, with higher scores indicating better physical and mental health functioning.^29^ The PAM-13 measures self-management ability, showing how ready, willing, and able individuals are to manage their health and health care and ranges from 0 (no activation) to 100 (high activation). The PAM-13 scores correspond to 1 of 4 levels of patient activation, ranging from low activation to high activation.^30^

### Statistical Analysis

Analyses followed the intention-to-treat principle. Descriptive information was presented as means with SDs or medians with IQRs for continuous variables and as counts and frequencies for categorical variables. Total attendance rates were calculated for each participant and subsequently allocated to one of the following groups: low attendance (0 to 10 sessions), medium attendance (11 to 20 sessions), or high attendance (21 to 30 sessions). We categorized participants with all measurements available as complete cases, those missing 1 follow-up measurement as semicomplete, and those with no follow-up measurements as dropouts to see whether characteristics of dropouts and semicompleters differed from completers.

First, differences between the lifestyle intervention and TAU groups in the primary and secondary outcomes between baseline and 3 (body weight only), 6, and 12 months were analyzed with linear mixed models.^31^ In these, time (categorical variable) and interactions between time dummy variables and groups were included. The multilevel hierarchical structure (measurements over time among clients clustered within FACT teams) was taken into account by adding random intercepts at the patient and FACT team levels. Two models were analyzed: (1) crude analyses with only an adjustment for baseline values (added as time-independent variables) and (2) adjusted analyses with additional covariates.^32^ Covariates were selected based on differences in baseline values between the lifestyle intervention and TAU groups and on whether covariates could be prognostic for the intervention’s outcomes.^33^ This resulted in adjustments for the following baseline covariates: sex, primary mental health diagnosis (schizophrenia or other psychotic disorder, bipolar disorder, depressive or anxiety disorder, personality disorder, autism spectrum disorder, or other psychiatric disorder), if the participant was in a relationship (yes or no), and smoking status (yes or no) at each time point. We checked relevant assumptions. The distribution of triglycerides was skewed and was therefore log-transformed, and the (back-transformed) regression coefficients were presented as ratios.

Additional analyses included crude and adjusted multivariable logistic regression analyses to assess proportion of weight changes (5% or more and 10% or more weight loss between baseline and 3, 6, and 12 months). Adjusted analyses included the following covariates: sex, primary mental health diagnosis, relationship (yes or no), and smoking status change between baseline and 3, 6, and 12 months (never smoked or started, stopped, or continued smoking). Furthermore, we performed a per-protocol analysis where we assessed whether intervention attendance (low attendance, medium attendance, and high attendance) influenced body weight change. Finally, additional post hoc analyses (not described in the protocol^16^) included linear mixed model analyses to assess overall effects over the 12 months to gain further insight into average differences between lifestyle intervention and control groups over the intervention period.^32^

Significance was set at *P* < .05, and all *P* values were 2-tailed. Analyses were performed using Stata version 16.0 (StataCorp).

## Results

### Patient Flow and Sample Characteristics

**Figure 1.**
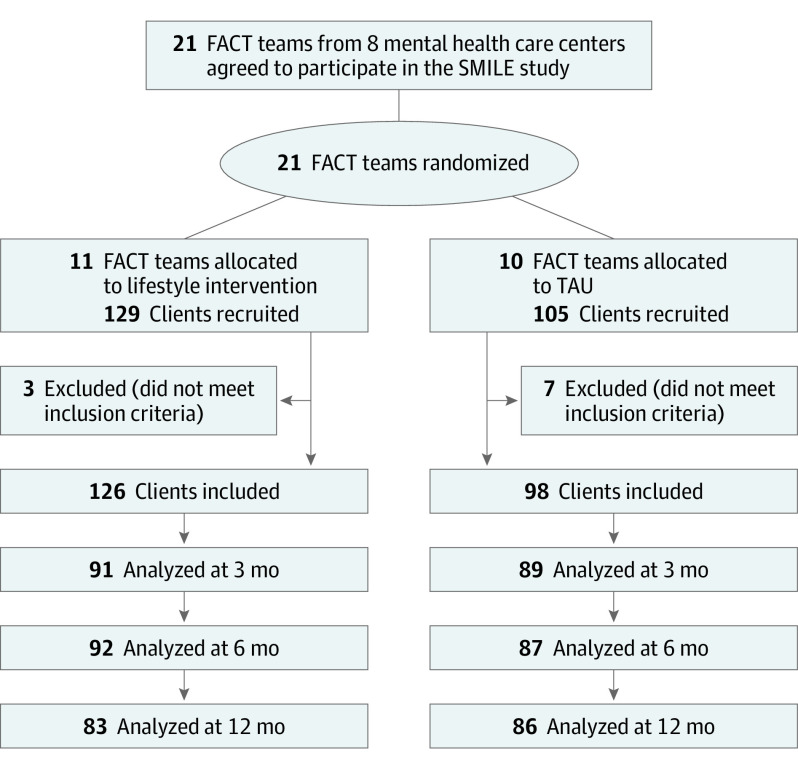
CONSORT Flow Diagram of Recruitment and Attrition for the Severe Mental Illness Lifestyle Evaluation (SMILE) Study Analysis samples are based on available measurements of primary outcome (body weight). As some participants missed a follow-up measurement but did participate in later measurements, not all numbers decrease within groups. FACT indicates flexible assertive community treatment.

The study population included 11 lifestyle intervention teams (126 participants) and 10 TAU teams (98 participants). Of 224 included patients, 137 (61.2%) were female, and the mean (SD) age was 47.6 (11.1) years. After randomization, 3 participants in the lifestyle intervention group and 7 in the TAU group were excluded for not meeting the inclusion criteria (Figure 1). In the lifestyle intervention group, there was a median (IQR) of 11 (10.5-13) participants per FACT team in the lifestyle intervention group and 10.5 (8.5-11.8) participants per FACT team in the TAU group. Data on the primary outcome were available for 122 participants (97%) in the lifestyle intervention group and 93 participants (95%) in the TAU group at baseline; 91 (72%) and 89 (91%), respectively, at 3 months; 92 (73%) and 87 (89%) at 6 months; and 83 (66%) and 86 (88%) at 12 months (Figure 1). Participants and case managers did not report any study-related harm. Characteristics for complete cases, semicomplete cases, and dropouts are reported in eTables 5 to 7 in Supplement 2.

Baseline characteristics are shown in Table 1. There were more women in the lifestyle intervention group (88 [70%]) compared with TAU (49 [50%]). In the lifestyle intervention group, fewer participants had schizophrenia or other psychotic disorders (53 [42%] vs 60 [61%]), more were in a relationship (38 [30%] vs 23 [24%]), and fewer were active smokers (43 [34%] vs 48 [49%]). Mean (SD) body weight (101.5 [19.6] vs 102.1 [17.7]) and BMI (35.4 [6.3] vs 34.2 [5.8]) were similar for both groups.

**Table 1.** Baseline Characteristics Among Lifestyle Intervention and Treatment as Usual (TAU) Group Participants

Characteristic	Mean (SD)	
	Lifestyle intervention (n = 126)	TAU (n = 98)
Demographic characteristics		
Age, y	47.6 (11.4)	47.6 (10.8)
Sex, No. (%)		
Male	38 (30)	49 (50)
Female	88 (70)	49 (50)
Self-reported nationality, No. (%)		
Dutch	118 (94)	87 (89)
Non-Dutch	4 (3)	8 (8)
Marital status, No. (%)		
Never married	66 (52)	53 (54)
Married	26 (21)	12 (12)
Divorced, separated, or widowed	29 (23)	28 (29)
In a relationship, No. (%)	38 (30)	23 (24)
Highest level of schooling, No. (%)		
Primary education	18 (14)	12 (13)
Secondary education	32 (25)	36 (37)
Vocational education	59 (47)	35 (36)
Higher education	13 (10)	11 (12)
Time receiving ambulatory care, y	8.4 (7.1)	11 (8.4)
Diagnosis		
Primary mental health diagnosis, No. (%)		
Schizophrenia or other psychotic disorder	53 (42)	60 (61)
Bipolar disorder	22 (17)	10 (10)
Depressive or anxiety disorder	12 (10)	8 (8)
Personality disorder	17 (14)	4 (4)
Autism spectrum disorder	5 (4)	4 (4)
Other psychiatric disorder	9 (7)	4 (4)
Somatic comorbidity diagnosis as reported by health care professionals, No. (%)		
Diabetes (type 1 or 2)	15 (12)	8 (8)
Hypertension	15 (12)	10 (10)
COPD	1 (0.8)	2 (2)
Clinical measurements		
Body weight, kg	101.5 (19.6)	102.1 (17.7)
Body mass index^a^	35.4 (6.3)	34.2 (5.8)
Blood pressure, mm Hg		
Systolic	133.9 (20.9)	129.1 (15.5)
Diastolic	85.1 (10.6)	81.9 (9.9)
Cholesterol, mg/dL		
Total	196.4 (41.5)	196.3 (39.4)
HDL	48.7 (13.8)	46.7 (12.4)
LDL	120.4 (40.4)	118.4 (34.5)
Triglycerides, median (IQR), mg/dL	141.6 (97.4-205.8)	159.3 (115.1-221.3)
Fasting glucose, mg/dL	111.8 (30.7)	110.3 (24.8)
Patient-reported measurements		
Active smokers, No. (%)	43 (34)	48 (49)
Average number of cigarettes	18.6 (8.9)	19.4 (10.7)
Perceived physical health status^b^	5.6 (1.9)	6.2 (1.9)
Perceived mental health status^b^	5.8 (2.0)	6.3 (2.3)
Perceived healthy PA pattern^b^	5.3 (2.1)	5.8 (2.3)
Perceived healthy nutrition pattern^b^	6.0 (2.0)	6.4 (2.0)
Average sleep in last 2 weeks, h	7.8 (2.1)	7.9 (2.2)
Satisfaction with weight^c^	2.3 (2.1)	3.7 (2.4)
Satisfaction with PA behavior^c^	4.7 (2.3)	5.5 (2.5)
Satisfaction with dietary behavior^c^	5.4 (2.2)	6.0 (2.3)
Satisfaction with sleep pattern^c^	5.5 (2.7)	6.0 (2.6)
SF-12 physical component summary^d^	40.1 (10.0)	45.3 (10.3)
SF-12 mental component summary^d^	40.6 (12.0)	43.0 (12.7)
PAM-13 score^e^	52.0 (10.0)	55.0 (12.8)
PAM-13 level^f^	2.0 (0.9)	2.2 (1.0)

Abbreviations: COPD, chronic obstructive pulmonary disease; HDL, high-density lipoprotein; LDL, low-density lipoprotein; PA, physical activity; PAM-13, Patient’s Activation Measure; SF-12, 12-item Short-Form Health Survey.

SI conversion factors: To convert cholesterol to mmol/L, multiply by 0.0259; triglycerides to mmol/L, multiply by 0.0113; glucose to mmol/L, multiply by 0.0555.

^a^ Calculated as weight in kilograms divided by height in meters squared.

^b^ Scored on a numerical scale ranging from very unhealthy (0) to very healthy (10) based on the last 2 weeks.

^c^ Scored on a numerical scale ranging from very dissatisfied (0) to very satisfied (10).

^d^ Scores range from 0 to 100, with a higher score indicating better physical or mental health functioning.

^e^ Scores range from 0 to 100, with higher scores indicating higher patient activation.

^f^ Levels range from 1 to 4, with levels 1 and 2 indicating low activation, level 3 indicating moderate activation, and level 4 indicating high activation.

### Outcomes

Adjusted effect sizes and 95% CIs for primary and secondary outcomes are shown in Table 2. Figures for the course of the 12-month intervention period for all unadjusted mean outcomes are visualized in the eFigure in Supplement 2. Crude effect sizes for all analyses and crude and adjusted results for clinically relevant weight loss are included in eTables 1 to 4 in Supplement 2.

**Table 2.** Adjusted Models at 3, 6, and 12 Months Compared With Baseline and Effects on Outcomes Over the 12-Month Follow-up Period^a^

Outcome	β (95% CI)			
	Baseline to 3 mo	Baseline to 6 mo	Baseline to 12 mo	Average difference^b^
Body weight change	−2.1 (−4.6 to 0.5)^c^	−2.4 (−5.3 to 0.4)	−3.3 (−6.2 to −0.4)	−2.8 (−5.5 to −0.1)
Body mass index change	NA	−0.8 (−1.8 to 0.2)	−1.1 (−2.1 to −0.1)	−1.0 (−1.9 to 0)
Systolic blood pressure	NA	1.9 (−2.2 to 6.0)	0.7 (−3.5 to 4.9)	1.2 (−2.2 to 4.6)
Diastolic blood pressure	NA	−1.1 (−3.8 to 1.5)	−0.6 (−3.3 to 2.2)	−0.9 (−3.2 to 1.4)
Total cholesterol	NA	NA	−0.3 (−0.6 to 0)	NA
HDL cholesterol	NA	NA	−0.1 (−0.1 to 0)	NA
LDL cholesterol	NA	NA	−0.3 (−0.6 to 0)	NA
Triglycerides	NA	NA	1.1 (1.0 to 1.3)^d^	NA
Fasting glucose	NA	NA	0.2 (−0.3 to 0.8)	NA
Perceived physical health^e^	NA	−0.1 (−0.7 to 0.4)	0.1 (−0.4 to 0.7)	0 (−0.5 to 0.5)
Perceived mental health^e^	NA	0.1 (−0.5 to 0.6)	0.1 (−0.4 to 0.7)	0.1 (−0.4 to 0.5)
Perceived healthy PA pattern^e^	NA	0.1 (−0.5 to 0.6)	0.2 (−0.4 to 0.8)	0.1 (−0.4 to 0.6)
Perceived healthy nutrition pattern^e^	NA	0 (−0.5 to 0.5)	0.1 (−0.4 to 0.6)	0 (−0.4 to 0.4)
Average sleep in last 2 wk	NA	−0.3 (−0.8 to 0.2)	−0.5 (−1.0 to 0)	−0.4 (−0.8 to 0)
Satisfaction with weight^f^	NA	1.0 (0.3 to 1.7)	0.4 (−0.3 to 1.1)	0.7 (0.1 to 1.3)
Satisfaction with PA behavior^f^	NA	0 (−0.6 to 0.7)	0.1 (−0.5 to 0.8)	0.1 (−0.5 to 0.6)
Satisfaction with dietary behavior^f^	NA	−0.1 (−0.6 to 0.5)	0.1 (−0.5 to 0.6)	0 (−0.5 to 0.5)
Satisfaction with sleep pattern^f^	NA	0.7 (0 to 1.4)	0.2 (−0.6 to 0.9)	0.4 (−0.1 to 1.0)
SF-12 physical component summary^g^	NA	0.5 (−2.3 to 3.4)	0 (−2.9 to 2.9)	0.3 (−2.2 to 2.7)
SF-12 mental component summary^g^	NA	−1.9 (−5.8 to 2.0)	−0.1 (−4.1 to 4.0)	−1.1 (−4.5 to 2.3)
PAM-13^h^	NA	1.8 (−2.0 to 5.7)	−1.6 (−5.6 to 2.3)	0.2 (−3.3 to 3.8)

Abbreviations: HDL, high-density lipoprotein; LDL, low-density lipoprotein; PA, physical activity; PAM-13, Patient’s Activation Measure; SF-12, 12-item Short-Form Health Survey.

^a^ Adjusted mixed models corrected for baseline sex, primary mental health diagnosis, relationship status, and time-dependent smoking status (yes or no).

^b^ Mean difference in outcome variable over time between the intervention and treatment as usual groups.

^c^ Corrected for baseline smoking status because smoking status was not measured at 3 months.

^d^ Regression coefficients represented as ratios because of log-transformed triglyceride levels.

^e^ Scored on a numerical scale ranging from very unhealthy (0) to very healthy (10) based on the last 2 weeks.

^f^ Scored on a numerical scale ranging from very dissatisfied (0) to very satisfied (10).

^g^ Scores range from 0 to 100, with higher scores indicating better physical or mental health functioning.

^h^ Scores range from 0 to 100, with higher scores indicating higher patient activation.

#### Body Weight Change

Participants in the control group gained a mean (SD) of 0.04 (7.1) kg after 12 months, while participants in the lifestyle intervention group lost a mean (SD) of 2.6 (8.4) kg after 12 months. Results from the adjusted mixed models (Table 2) are as follows: from baseline to 3 months, compared with TAU, the lifestyle intervention group lost 2.1 kg (95% CI, −4.6 to 0.5) more; from baseline to 6 months, the lifestyle intervention group lost 2.4 kg (95% CI, −5.3 to 0.4) more; and from baseline to 12 months, the lifestyle intervention group lost 3.3 kg (95% CI, −6.2 to −0.4) more. The decrease in BMI from baseline to 12 months was significantly larger for the lifestyle intervention group than for the TAU group (−1.1; 95% CI, −2.1 to −0.1).

#### Clinical Outcomes

The lifestyle intervention generally had little or no effect on diastolic blood pressure, total cholesterol level, high-density lipoprotein cholesterol level, low-density lipoprotein cholesterol level, triglyceride level, and fasting glucose level (Table 2).

#### Patient-Reported Outcomes

No statistically significant or clinically relevant differences were found between the lifestyle intervention and TAU groups after 6 and 12 months for self-assessed physical health, mental health, physical activity pattern, nutrition pattern, satisfaction with weight, physical activity, dietary and sleep behavior, average hours of sleep in the last 2 weeks, quality of life (SF-12), and patient activation measure (PAM-13) (Table 2).

#### Weight Change and Attendance at Intervention Sessions

During the 12-month intervention period, participants attended a median (IQR) of 16 (5-23) of the 30 sessions offered. A total of 45 participants (36%) had low attendance, 34 (27%) had medium attendance, and 47 (37%) had high attendance.

**Figure 2.**
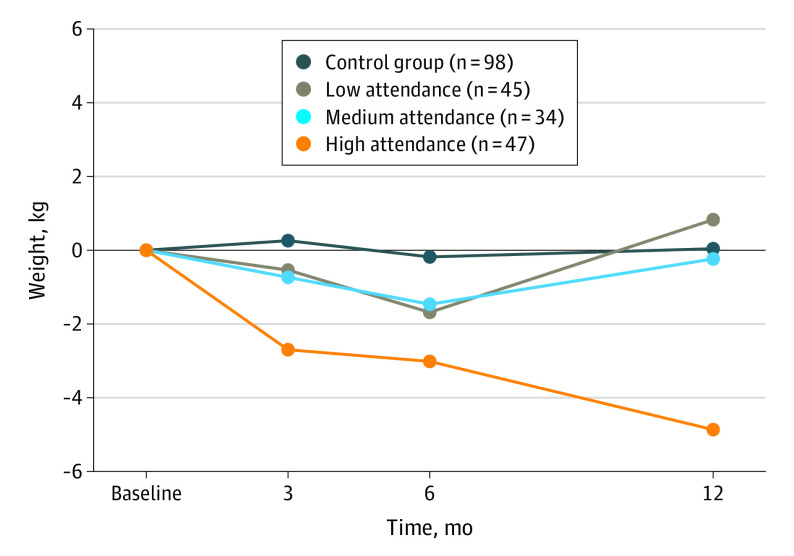
Weight Change Over the 12-Month Period by Attendance

For the per-protocol analyses, the course of weight change over the 12-month period for lifestyle intervention participants with low, medium, and high attendance and for controls are presented in Figure 2. Low attenders started the lifestyle intervention with higher mean (SD) baseline weight than medium and high attenders (low, 109.1 [20.2] kg; medium, 96.7 [18.1] kg; high, 98.3 [18.5] kg). On average, low attenders gained a mean (SD) of 0.8 (8.3) kg after 12 months, medium attenders did not change (−0.2 [7.8] kg), and high attenders lost −4.9 (8.1).

## Discussion

The group-based lifestyle intervention resulted in −3.3 kg (95% CI, −6.2 to −0.4) greater body weight loss than TAU after 12 months. Participants with higher attendance rates in the lifestyle intervention lost more weight than those with medium or low attendance rates. However, with small subgroups, these findings need confirmation in future studies. We found no differences for secondary outcomes other than BMI (−1.1; 95% CI, −2.1 to −0.1).

Body weight differences between lifestyle intervention and TAU groups after 6 months were smaller (−2.4 kg; 95% CI, −5.3 to 0.4) than for the STRIDE study (−4.4 kg) but were larger after 12 months (−3.3 kg [95% CI, −6.2 to −0.4] vs −2.6 kg, respectively).^19^ In addition, the reduction in weight found after 12 months in our study (−3.3 kg) is greater than in a meta-analysis analyzing lifestyle interventions for weight management in people with SMI.^14^ This meta-analysis of 32 studies found a pooled effect of −2.2 kg.^14^ Similarly, in a 2021 meta-analysis^34^ including 24 studies analyzing inpatient and outpatient lifestyle interventions for diet and exercise and their effect on physical and psychological health in schizophrenia spectrum disorders and first episodes of psychosis, a pooled weight reduction effect of 2.1 kg was found at the end of the interventions. Furthermore, the ACHIEVE study showed 2.5 kg (95% CI, −4.1 to −0.8) weight reduction compared with controls after 12 months and 3.2 kg (95% CI, −5.1 to −1.2) after 18 months. A possible explanation for the fact that the SMILE study has resulted in better outcomes compared with other studies is that the broad capability of FACT teams to provide assertive outreach in the community may have resulted in greater support for making lifestyle changes for participants in the lifestyle intervention group.

We did not find an effect of the lifestyle intervention on quality of life, in line with results from other lifestyle interventions.^35^ Finally, research suggests that lifestyle interventions may have positive effects on depression and anxiety severity,^35^ which were not measured in this study. Future researchers should consider more patient-reported outcomes as they are beneficial to people with SMI. This study indicated a dose-response effect, as participants with high attendance rates lost more mean (SD) weight (−4.9 [8.1] kg) than those with low (0.8 [8.3] kg) or medium (−0.2 [7.8] kg) rates, but because groups were small, we cannot draw strong conclusions.

We must realize that achieving large effects within the total group of patients is not realistic. In the above-mentioned meta-analyses,^14^ average weight loss of groups was presented. However, knowing that there are large differences within groups, some patients will benefit strongly from the lifestyle intervention while others will benefit less or not at all. In this regard, the similarities between the general population and our study population will likely outweigh the differences. Motivation to change lifestyle behaviors is an important factor, which in turn determines participation in the lifestyle intervention. It is therefore important not only to base the practical value of the lifestyle intervention on group averages but also to define in more detail the subgroups that do or do not benefit from the intervention and to analyze the influencing factors in more detail. Here lies an important challenge for future research. The results of these analyses contribute to the development of strategies to better tailor lifestyle interventions to patients’ characteristics and preferences. We recommend more research to gain in-depth knowledge on how lifestyle interventions can be better tailored for this population to better meet their interests and needs, increase attendance rates, and increase benefits.

A broad approach is needed in which the obesogenic living environment of people with SMI is also improved (such as introducing healthy facilities in mental health institutes and changing governmental policies, such as lowering value-added taxes on fruit and vegetables), since unhealthy living environments, low socioeconomic status, and loneliness have big impacts on lifestyle behavior.^36,37,38,39,40,41^ Changing lifestyle is even more difficult if the living environment discourages healthy behavior or even stimulates unhealthy behavior. Additionally, it is important that mental health institutes (including FACT teams) improve integration of a healthy lifestyle (eg, physical activity, healthy food) into their daily clinical practice. If lifestyle becomes an integral part of the treatment program, this can support clients to make sustainable improvements in their physical health status.

### Strengths and Limitations

This study has several strengths. It was performed in a pragmatic, real-world setting in 21 FACT teams at 8 Dutch mental health centers. This improved its external validity because we made use of the available time, resources, and staff of FACT teams comparable in scope with Dutch care outside this experimental context. This may positively influence the implementation of lifestyle intervention following this study. FACT care is being adopted in many other countries, with a comprehensive focus on symptomatic, functional, and personal recovery. Therefore, the FACT team setting offers ample opportunities for lifestyle interventions. To better understand our findings, we performed a process evaluation (reported separately^27,28^). It provided us with important details about the barriers to and facilitators of the lifestyle intervention, as perceived by both clients and health care professionals.

Our trial has limitations. The sample size was smaller than planned (224 participants instead of the anticipated 260). Despite a smaller-than-anticipated recruitment, we found a statistically significant difference in the primary outcome. A larger sample size would have resulted in a more precise effect estimate (smaller 95% CIs) but would probably not have changed the estimate itself. The SMILE study was an ambitious trial in this population, with the inclusion of 224 people with SMI within 1 year, with an almost complete range of sessions offered by professionals (98%) and with a patient participation rate of 52%.^27^ However, compared with other trials, our retention rates for the lifestyle intervention group were higher; for example, the STEPWISE study had a loss to follow-up of 19%.^15^ Dropout rates differed between groups (34% in SMILE vs 12% in TAU at 12 months), with dropouts in the lifestyle intervention group having a higher mean (SD) body weight at baseline (103.8 [17.8] kg vs 100.3 [20.4] kg), possibly causing an overestimation of the effect (eTables 5 to 7 in Supplement 2). It is difficult to provide suggestions for possible effective strategies to improve retention as some strategies that have shown to work well for a particular population may not work for others. Yet financial incentives, abridged questionnaires, and prenotifications have shown a positive effect on retention rates in mental health trials.^42^

Another limitation is the limited validity of the lifestyle behavior measurements. We chose not to measure lifestyle behavior using extensive questionnaires or objective measures because we wanted to avoid the risk of overburdening participating clients. Further, as it was not possible to blind participants to the intervention allocation and not feasible to blind measurement assessors, it is possible this may have biased results.

## Conclusions

This randomized clinical trial evaluated a 1-year group-based lifestyle intervention in a FACT team setting in the Netherlands. The group-based lifestyle intervention significantly reduced weight from baseline to 12 months in adults with SMI and overweight or obesity. Participants with higher attendance rates seemed to benefit most, but this subgroup was small, and these findings need confirmation in future research. The lifestyle intervention could be a useful tool for further implementation of lifestyle interventions by FACT teams.

## Notes

Supplement 1. Trial Protocol

Supplement 2. **eMethods.** Overview Intervention Content **eTable 1.** Crude Analyses of Primary and Secondary Outcomes **eTable 2.** Adjusted Analyses of Primary Outcome per Covariate **eTable 3.** Crude Analyses of Clinically Relevant Weight Loss **eTable 4.** Adjusted Analyses of Clinically Relevant Weight Loss **eTable 5.** Total Characteristics of Complete Cases and Dropouts **eTable 6.** Characteristics of Complete Cases and Dropouts in the Intervention Group **eTable 7.** Characteristics of Complete Cases and Dropouts in the TAU Group **eFigure.** Unadjusted Mean Differences Over the 12-Month Period for Primary and Secondary Outcomes
eReferences.

Supplement 3. Data Sharing Statement
