# Supplementary material for: Effectiveness of a Lifestyle Intervention for People With a Severe Mental Illness in Dutch Outpatient Mental Health Care: A Randomized Clinical Trial
Source: JAMA Psychiatry. 2023 Jun 21;80(9):886–94. doi: 10.1001/jamapsychiatry.2023.1566 (PMC10285675; doi:10.1001/jamapsychiatry.2023.1566)
Supplement: Supplement 1. — Trial Protocol [file jamapsychiatry-e231566-s001.pdf]

RESEARCH PROTOCOL

Medical Ethics Committee at VU University Medical Centre, Amsterdam

Ethical approval for this study was obtained from the Medical Ethical Committee of the VU University Medical Center in Amsterdam, The Netherlands (NL60315.029.17, registration number 2017.418).

Title: Cost-effectiveness of a tailored lifestyle intervention in patients with serious mental illness: the SMILE study

Coordinating investigator/project leader *Dr. Marcel Adriaanse* (*marcel.adriaanse@vu.nl*)

SMALL CHANGES MADE TO THE ORIGINAL PROTOCOL

Formatted: Num

Sample size: We planned to include 260 participants taking into account 20% drop-out, but finally included 224 participants in the study. It is unlikely that 36 extra participants would substantially change our results and conclusion. A larger sample size would have resulted in a more precise effect estimate (smaller 95% CI) but would probably not have changed the estimate itself.

Participating FACT-teams: Initially we planned on including 18 FACT-teams, but a total of 21 FACT-teams joined the study.

INTRODUCTION AND RATIONALE

Cardiovascular disease is one of the leading causes of the estimated 20-25 years reduced life expectancy for persons with serious mental illness (SMI) [1-6]. This excess cardiovascular mortality is primarily attributable to obesity, diabetes, hypertension, dyslipidemia and lifestyle factors [7]. Additionally, cardiovascular disease in persons with SMI contributes to enormous societal costs. In the Netherlands, €19.6 billion were spend on the treatment of mental disorders (22% of the total spend on care in 2011), cardiovascular disease comes in second place with €8.3 billion (). The reduction of these cardiovascular risks has been associated with performing lifestyle interventions [8-12].

Lifestyle intervention programs are the basis of efforts in assisting persons with SMI to improve somatic health. Most lifestyle intervention programs focus on reduction of weight and reduction of cardiovascular risks such as high cholesterol values, primarily by the means of improving physical activity and stimulating a healthy diet. For persons with SMI, such lifestyle intervention programs are the basis of efforts in assisting them with the reduction of cardiovascular risks. There is abundant evidence from systematic reviews and meta-analyses showing that these lifestyle interventions are effective in reducing cardiovascular risks in the general medical population [13], in people with type 2 diabetes [9, 11], and in persons with SMI [8, 12]. Recently, three randomized clinical trials published in high-impact journals showed that lifestyle interventions for persons with SMI are effective in reducing weight and cardiovascular risks in comparison with usual care [10, 14, 15].

However, whether a tailored lifestyle intervention is cost-effective compared with usual care in persons with serious mental illness in outpatient psychiatric treatment settings (so called Flexible Assertive Community Treatment teams) in the Netherlands has not been established yet. Therefore, this study aims to evaluate the cost-effectiveness of a tailored lifestyle intervention in persons with SMI in outpatient treatment settings (the SMILE study).

ADDED VALUE AND RELEVANCE FOR PRACTICE

Although there is strong evidence that lifestyle interventions are effective in patients with SMI, cost-effectiveness has not yet been established. Given the (financial) burden of morbidity and premature mortality related to unhealthy lifestyle behaviours in persons with SMI, evidence-based interventions for reducing (in particular) cardiovascular risk and associated costs are urgently needed. This is even more important because in research persons with SMI are typically excluded from lifestyle interventions targeted at the general population. Given the large number of persons with SMI and cardiovascular risks, and the associated high burden for the patient, family members and society, this study is a matter of urgency. This proposed research has substantial societal value as it provides financing organizations and governments with better insights how to spend the available resources in

the most efficient way. This urgent need is supported by recommendations from the European Psychiatric Association (EPA), the European Association for the Study of Diabetes (EASD) and the European Society for Cardiology (ESC), which aim to improve the care of persons suffering from a serious mental illness [7].

The SMILE project is based on the American STRIDE lifestyle intervention [10], developed for persons with SMI and effective in reducing weight and cardiovascular risk factors. The SMILE project will be carried out by trained mental health nurses working in FACT-teams in the Netherlands. It is hypothesized that the SMILE intervention is more effective than usual care in reducing weight, reduce cardiovascular risk factors, promote lifestyle behaviours, improve quality of life, health related self-efficacy and reduce health care costs.

OBJECTIVES

Primary Objective: To reduce weight after 1 year in persons with SMI in outpatient psychiatric treatment settings who are treated by Flexible Assertive Community Treatment teams (FACT-teams).

Secondary Objective(s): To reduce cardiovascular risks (central obesity, lipids, blood pressure, glucose), promote lifestyle behaviours, improve quality of life, health related self-efficacy and reduce health care costs after 1 year in persons with SMI in outpatient psychiatric treatment settings who are treated by FACT-teams.

STUDY DESIGN

The study will consist of an economic evaluation alongside a cluster randomized controlled trial using a 1 year follow-up. Effectiveness and cost-effectiveness of the tailored lifestyle intervention will be evaluated in comparison with usual care. Before the persons with SMI are recruited, participating FACT-teams will be randomly allocated to the tailored lifestyle intervention or usual care.

The study will be carried out in two parts. At first, half of the centres will start with the trial and about 6 months later, the other centres will start. The tailored lifestyle intervention lasts 12 months in total. Including inclusion of participants, analysis and reporting phase, the total duration of the SMILE study will be 48 months.

JUSTIFICATION STUDY DESIGN

Randomization will be performed at the level of the FACT teams. All persons with SMI receive the same treatment within a cluster (FACT team). Major reason for this randomization procedure is to avoid contamination of the treatment within the same FACT team. It is not possible to blind persons with SMI and FACT team professionals to the allocated intervention due to the nature of the tailored lifestyle program. However, the pragmatic design of the study enhances the external validity. Staff from FACT teams in the usual care control group will not be trained to perform the tailored lifestyle intervention until after the end of the trial. That way, FACT team members cannot unintentionally apply aspects of the intervention to their usual care for the SMI patients.

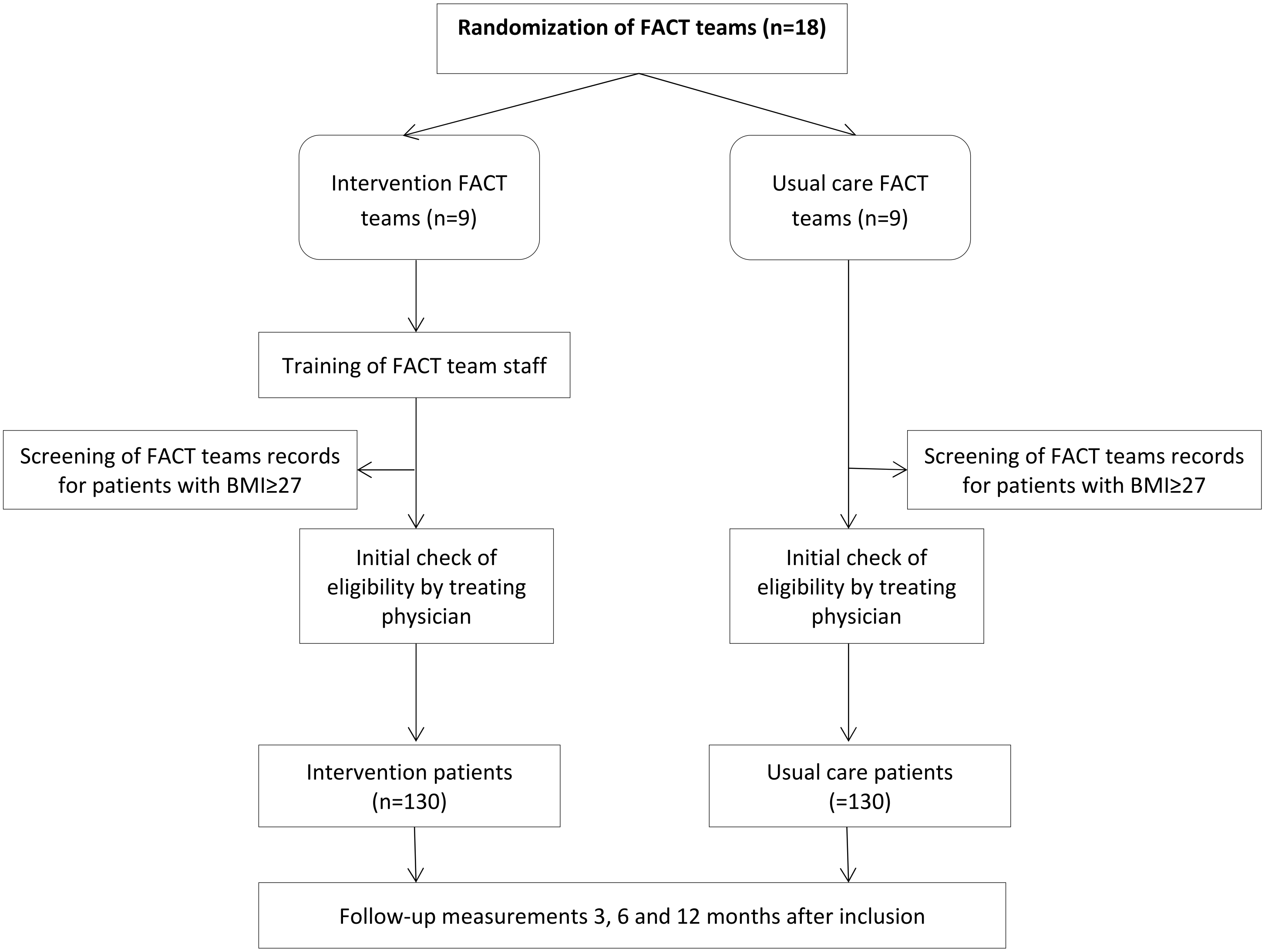
Randomization of FACT teams (n=18)
Intervention FACT teams (n=9)
Usual care FACT teams (n=9)
Training of FACT team staff
Screening of FACT teams records for patients with BMI≥27
Screening of FACT teams records for patients with BMI≥27
Initial check of eligibility by treating physician
Initial check of eligibility by treating physician
Intervention patients (n=130)
Usual care patients (=130)
Follow-up measurements 3, 6 and 12 months after inclusion

STUDY POPULATION

Population (base)

SMI is defined according to the consensus paper by Delespaul and the consensus group SMI [16]. Persons have SMI when: - there is a psychiatric disorder which requires long-term treatment and care (symptomatic not in remission); - and which is accompanied by severe impairment in social interaction and / or social functioning (non-functional remission); - the severe impairment is the cause and effect of a mental illness; - it is structurally c.q. a long time, at least several years; - coordinated care from professionals in care networks is indicated to realize the treatment plan. The consensus group also stated that SMI cannot be limited to certain psychiatric diagnoses. The most prevalent psychiatric disorders in the group of patients with SMI are: schizophrenia and related psychotic disorders, mood disorders (including bipolar disorder), severe personality disorders and addition disorders.

Adults (≥18 years) with SMI in care by Flexible Assertive Community Treatment (FACT) teams in the Netherlands will be included. The study aims to recruit 260 patients in total (for more information regarding the power calculation, see chapter 3.4). The prevalence of persons with SMI is 1.3% (n=216.000) of the total population and 1.6% (n=160.000) of the population of ≥18 and 65 years in the Netherlands in 2013. About 75% is cared for by the GGZ (Mental Health and Addiction Care institutes) or other providers [16]. In the Netherlands there are approximately 250 certified FACT-teams.

Inclusion criteria

In order to be eligible to participate in this study, a subject must meet all of the following criteria:

- patient with SMI
- age ≥18
- body mass index ≥27
- willing to and able to sign informed consent (mentally competent)

Exclusion criteria

A potential subject who meets any of the following criteria will be excluded from participation in this study:

- Contra-indications (to be assessed by the treating physician/psychiatrist) for participation due to acute psychiatric crisis or somatic diseases (e.g. bariatric surgery, cancer, heart attack or stroke)
- Subjects with a cognitive impairment sufficient to interfere with their ability to provide informed consent, complete study questionnaires, or participate in a group intervention.

- Women who are pregnant, breastfeeding, or planning a pregnancy during the course of the study
- Subjects not able to communicate in the Dutch language

Sample size calculation

This trial is powered to detect a mean difference of 4 kilograms in weight reduction after 1 year between the intervention and usual care group [10, 17]. This difference is based on the outcomes of multiple clinical trials including STRIDE, ACHIEVE and PREMIER [10, 14, 15, 17, 18]. using a power of 0.80, an alpha of 0.05 and an SD of 10 two groups of 100 patients are needed to detect a difference of 4 kilograms. Assuming an ICC of 0.01 for clustering of persons with SMI within a FACT-team, and a dropout rate of 20% [10, 14, 15], the aim is to recruit 260 patients in total.

TREATMENT OF SUBJECTS

Investigational treatment

In the Netherlands patients with SMI can be treated by FACT-teams. FACT opts to provide services to the whole group of people with SMI and provide long-term care for people with SMI who are not in psychiatric hospitals. Care by FACT includes management of illness and symptoms (treatment), guidance and practical assistance with daily living, rehabilitation and also recovery support. FACT teams are multidisciplinary teams which can include psychiatrists, nurses, psychologists, employment specialist, peer support workers and social workers.

THE INTERVENTION

The intervention is primarily modelled after the successful STRIDE intervention [10]. The STRIDE lifestyle intervention, was developed for persons with SMI and was effective in reducing weight and cardiovascular risk factors. The STRIDE intervention was based on prior research (PREMIER clinical trial) [19], behaviour change theories such as the trans theoretical model [20, 21] and motivational theory [22-24]. The basis of the STRIDE intervention will be used in the SMILE study. Additionally, when included patients are current smokers, attention is given to smoking cessation. This was not included in the original stride intervention. Patients who want to quit smoking will be directed to their general practitioner or treating physician. They will be offered standard smoking cessation care that is frequently offered.

The SMILE intervention will be carried out by health care workers the FACT-teams. This could include nurses, health care workers, peer support workers or similar disciplines.
The proposed tailored lifestyle intervention lasts 12 months and consists of (A) the initial intervention (first 6 months), and (B) the maintenance phase (second 6 months).

(A) *The initial intervention:* the core is a series of weekly 2-hour group meetings delivered over 6 months. The initial intervention consists of 24 sessions in total. The outline for the initial intervention sessions is presented in Table 1. Participants are further taught to keep records of 1) food, beverages, and calories consumed; 2) servings of fruits, vegetables, and low-fat dairy products; 3) fiber and fat intake; 4) daily minutes exercised and 5) nightly hours slept. Goals included are ≥ 25 minutes of moderate physical activity per day, primarily through walking; increased fruit, vegetable, low-fat dairy consumption and improved sleep quality. Participants will receive a workbook to support them to achieve a healthy calorie, fat, and carbohydrate intake. The intervention relies on engaging sessions and small-group activities to facilitate program participation, practice of behavioural self-management, the use of problem-solving skills, and to foster social support and program ownership. Core components are increasing awareness of health-related practices through self-monitoring, creating

personalized plans, reducing energy intake by reducing portions, increasing consumption of low-calorie density foods, increasing physical activity, managing high-risk eating situations, and addressing effects of mental health on change efforts.

Table 1. Outline SMILE initial intervention sessions

Session 1: Welcome and Introduction to SMILE
Session 2: Portion Control
Session 3: Introduction to Energy Balance and Goal Setting
Session 4: Breakfast and Regular Meals
Session 5: Working Toward a Healthier Diet
Session 6: Eating Healthfully on a Budget
Session 7: Are You on Target? Progress Check
Session 8: Planning Ahead for Meals
Session 9: Social Systems and Social Support
Session 10: Medication Side Effects and Weight Gain
Session 11: Stress Management and Sleep
Session 12: Are You on Target? Progress Check, Reframing Negative Self-Talk
Session 13: Conscious Eating
Session 14: Overeating, Emotional Eating, and Binge Eating
Session 15: Dining Out
Session 16: Importance of Physical Activity
Session 17: Revisiting Meal Planning and Portion Control
Session 18: Are You on Target? Progress Check, Motivation and Problem Solving
Session 19: Planning for Future Social Support
Session 20: Beyond Triggers
Session 21: Managing Plateaus
Session 22: Exercising to Maintain Weight Loss
Session 23: Planning for Changes in Mental Health Status
Session 24: Celebrating Accomplishments

(B) *The maintenance phase.* The maintenance phase includes 6 months of group sessions that take place once per month focusing on maintaining weight loss, in particular through the application of problem solving techniques and motivational enhancement. Sessions will be supplemented with monthly individual telephone sessions (about 15 minutes) and email (3-4 exchanges) if needed with group leaders. Contacts will be collaborative, discussing lifestyle change efforts, with guided problem solving.

COMPARISON: TREATMENT AS USUAL

The usual procedures of care for patients will be given. These procedures are described in the Dutch guidelines for lifestyle in patients with SMI published in 2015 [25]. However, not all centres follow these guidelines to their full extent. FACT-teams delivering the standard care will not receive any training for the trial. If results of the present study indicate that the lifestyle intervention is effective, FACT-teams in the usual care group will be offered the training. Agreements on the delivery of usual care by FACT-teams during the study period will be formalized in a contract.

TRAINING

Two healthcare workers of each FACT team delivering the intervention will be trained. They will function as role models for their colleagues within the FACT team on the subject of lifestyle for patients with SMI. They will receive a training consisting of: (a) consequences of an unhealthy lifestyle for patients with SMI and medication side effects, (b) basics of motivational interviewing and (c) all components of the lifestyle intervention (including contents of all group sessions).

METHODS

Study parameters/endpoints

All study parameters are measured at baseline before the intervention starts and after 12 months, unless otherwise indicated in paragraph 8.3.

With each centre an (if possible) independent assessor will perform the measurements. Assessors are trained by the research team.

Main study parameter/endpoint

PRIMARY OUTCOME

Weight loss

Weight (measured in kg) is measured by assessors using calibrated scales. Participants will wear light clothing (i.e. with empty pockets, no belt) and no shoes or jackets. The time during the day weight was measured will be reported.

Secondary study parameters/endpoints (if applicable)

Central obesity, height, body mass index (BMI), systolic and diastolic blood pressure, LDL, HDL, total cholesterol, triglycerides, glucose metabolism, quality of life (SF12 and EQ-5D-5L) and health related self-efficacy (PAM).

GENERAL STUDY PROCEDURES

Central obesity

Waist circumference (central obesity) will be measured by in centimetres in duplicate using a flexible non-stretching tape halfway between the iliac crest and lowest rib in standing position at the end of an expiration.

Height

Height will be measured to the nearest 0.5 cm without shoes using a tape line. Height is only measured at baseline.

Body mass index (BMI)

BMI will be computed as: bodyweight (kg) divided by height (m^2).

Systolic and diastolic blood pressure

Defined in mmHg and will be measured in seated position with legs uncrossed after at least five minutes of rest, and then again after an additional 30 second rest period using a blood pressure monitor. In case of abnormal values, the general practitioner or head practitioner will be contacted. Permission for this is included in the informed consent form. Assessors of the systolic and diastolic blood pressure will report at what time during the day the blood pressure was measured.

LABORATORY MEASUREMENTS

Serum **LDL**, **HDL**, **total cholesterol**, **triglycerides, glucose metabolism** will be measured by the according laboratories patients go to for their annual check-ups. All lab measurements will be collected after an overnight (8–12 hour) fast. For each lipid parameter blood samples will be taken by means of venapunction. Each laboratory will use their own according validated essays. Abnormal values will be contacted with treating general practitioners. For these measurements two samples will have to be collected (one sample of 4ml EDTA and one sample of 3ml heparin blood).

PATIENT-REPORTED QUESTIONNAIRES

All participants will complete questionnaires online or by paper and pencil together with an assessor trained by the research team.

Quality of life

Quality of life will be measured using Short Form-12 (SF-12). The SF-12 is a generic, reliable and validated instrument, containing 12 items derived from the Short Form-36 questionnaire [26]. The physical and mental component summary scores of the SF-12 will be used. Dutch age- and sex-standardized population norms are available [27].

Additionally, the EQ-5D-5L will be used to compute quality adjusted life years. The EQ-5D-5L rates self-care, mobility, pain, psychic functioning (anxiety/ depression), and usual activities on a 5-point scale. The patients' EQ-5D-5L health states will be converted into utility scores using the Dutch tariff. QALYs will subsequently be calculated using linear interpolation between measurement points.

Health related self-efficacy

The Patient Activation Measure (PAM-13) is a reliable questionnaire which contains 13 items derived from the original PAM-22 [28]. The questionnaire assesses patients' self-reported knowledge, skills and confidence for health related self-efficacy.

COSTS

Costs questionnaire

Cost questionnaires will be completed at baseline, after 6 months and after 12 months. Questions are

based on the TiC-P [29] and will include health care utilization, work absenteeism and presenteeism, and sports. Mental health ward admissions and medication use, including medications taken that affect weight, mood stabilizer medication use and anti-depressant medication use will be used as well. Information regarding medication use is used from the pharmacy of the Patient. Of this patients are informed in the patient information letter and will be asked to give their pharmacy information to the assessor.

Other study parameters (if applicable)

DEMOGRAPHIC DATA

Demographic data (ethnicity, age, gender, marital status, employment status, education, diagnosis of SMI, time in care in FACT-team) will be collected directly from the patient records.

SMOKING STATUS

Patients will be asked if they are current smokers and, if so, how many cigarettes they smoked in the last 7 days. Additionally, at 6 months and 12 months follow-up measurements patients who mentioned to be current smokers will be asked if they participated in a smoking cessation program (such as a smoking cessation intervention) or another kind of help for smoking cessation (such as guidance by a caregiver or physician).

LIFESTYLE FACTORS

Physical activity and nutrition status will be asked by one question using a VAS-scale concerning the past 4 weeks.

COMPLIANCE

During the intervention phase intervention appointments not attended (no-shows) will be assessed by the staff of the according FACT-Team.

Randomisation, blinding and treatment allocation

RANDOMISATION

Before persons with SMI are recruited, participating FACT-teams will be randomly allocated to serve as intervention condition (where the tailored lifestyle intervention will be implemented) or control FACT-teams (where care as usual will be given). Patients will be allocated to either one of the treatment conditions, based on the FACT-teams that treated them.

Randomisation will be stratified by centers. This is done to ensure the comparability between FACT-teams and therefore reduce bias. The randomization results will be communicated to the participating FACT-teams by the researchers.

Randomisation will be performed by a statistician blinded to the characteristics of the FACT-teams using a computer generated list of random numbers.

BLINDING

Blinding of patients and FACT-team members is not possible due to the nature of the intervention. The statistician and health economist will be blinded for the allocation of the intervention by keeping the information of the allocation of the intervention in a separate data file.

The assessors of measurements will also not be blinded, because if they are employed by the FACT-team they will know whether the fact team provides the lifestyle intervention or not.

Study procedures

Assessments will be made according to the schedule listed below.

Registry Schedule of Assessments

	**Baseline**	**3 months**	**6 months**	**12 months**
**DEMOGRAPHICS**				
Age, ethnicity, gender	x			
Height (cm)	x			
Weight (kg)	x	x	x	x
Marital status	x			x
Education level	x			x
Employment status	x			x
Diagnosis of SMI	x			x
**PRIMARY OUTCOME**				
Weight loss	x	x	x	x
**ANTHROPOMETIC MEASURES**				
BMI (kg/m^2)	x	x	x	x
Central Obesity (cm)	x	x	x	x
**PATIENT REPORTED QUESTIONNAIRES**				
SF12	x		x	x
PAM	x		x	x
Smoking status	x		x	x
**COSTS**				
Cost questionnaire	x		x	x
**CLINICAL MEASUREMENTS**				
HDL Cholesterol (mmol/l)	x			x
LDL Cholesterol (mmol/l)	x			x
Total Cholesterol (mmol/l)	x			x
Triglyceride (mmol/l)	x			x
Glucose metabolism (mmol/l)	x			x
Systolic BP (mm/hg)	x		x	x
Diastolic BP (mm/hg)	x		x	x
**OTHER**				
Intervention appointments not attended	Weekly /monthly			
Prescribed medication	x		x	x
Admission Mental health ward	x		x	x

STATISTICAL ANALYSIS

Characteristics of the patients in the treatment groups will be presented using descriptive statistics (mean (standard deviation), median (range) or (proportion)) to determine whether randomization was successful; i.e., to assess if balanced groups were obtained after randomization. All analyses will be on an intention-to treat basis.

Primary study parameter(s)

DATA-ANALYSIS AND PRESENTATIONS/SYNTHESIS

Differences between the tailored lifestyle intervention group and the usual care group will be tested using mixed methods analyses. To test the hypothesis that the tailored lifestyle intervention is more effective than usual care in reducing weight loss (primary outcome) over time, linear mixed models will be used. The obtained beta's describe the reduction in kilograms in the intervention group relative to the control group. Mixed model analyses can correct for clustering of data within the participating centres by adding "levels" to the analysis. The main analysis will consist of fully corrected models, which will be corrected for significant differences between the two groups at baseline. We will also evaluate whether dose, defined as the number of intervention sessions and number of telephone contacts, is related to change in the outcomes across time among participants who receive the intervention. We will include gender, age, and other variables that are known to affect weight as covariates in all analyses.

Secondary study parameter(s)

Secondary outcomes will be analysed as the primary data analysis. If necessary, logistic mixed models will be performed instead of linear mixed models depending on the outcome.

Other study parameters. Economic evaluation. Process evaluation.

Other parameters are presented as follows: ethnicity (categorical), age (continuous), gender (dichotomous), diagnosis of SMI (categorical), completed education (categorical), marital status (categorical) and employment status (categorical).

COST EFFECTIVENESS ANALYSIS

General considerations

Apart from statistical analysis, an economic evaluation will be performed alongside the trial. The aim of the economic evaluation is to relate the difference in costs between the two treatment conditions to the difference in clinical effects. Both a cost-effectiveness (CEA) and a cost-utility (CUA) will be performed from a societal and healthcare perspective according to Dutch guidelines [30]. The time horizon of the economic evaluation is 12 months, thus discounting is not necessary. Sensitivity

analyses will be performed to assess the robustness of the results using different assumptions regarding costs and effects. For the measurement and valuation of the costs the Dutch costing guidelines will be used [30].

Costs analysis

Costs will be measured using questionnaires based on the TiC-P [29] adapted to SMI patients group at baseline, 6 and 12 months of follow-up. Cost data include costs of the intervention, other health care utilization, patient and family costs, costs of productivity losses and costs for performing sports. Quality adjusted life years will be calculated using the EQ-5D-5L [31] with Dutch reference values. Uncertainty around incremental cost-effectiveness and cost-utility ratios will be estimated using bootstrapping techniques and graphically presented on cost-effectiveness and cost-utility planes. Bootstrapping will be used to estimate 95% confidence intervals around cost-difference per group.

Patient outcome analysis

The following effect measures will be included in the in the economic evaluation:
1) decrease in weight (kg);
2) improvement of cardiovascular risks (central obesity, lipids, blood pressure, glucose);
3) quality-adjusted life-years based on the Dutch tariff for the EQ-5D-5L

Cost-effectiveness and cost-utility analysis

The analysis will be done according to the intention-to treat principle. Missing cost and effect data will be imputed using multiple imputation according to the MICE algorithm developed by van Buuren [32]. Rubin's rules will be used to pool the results from the different multiple imputed datasets. Incremental cost-effectiveness ratios (ICERs) will be calculated by dividing the difference in the mean total costs between the treatment groups by the difference in mean effect between the treatment groups. Bias-corrected and accelerated bootstrapping with 5000 replications will be used to estimate statistical uncertainty surrounding the ICERs. Uncertainty surrounding ICERs will be graphically presented on cost-effectiveness planes. Cost-effectiveness acceptability curves will also be estimated using the net benefit framework [33]. Cost-effectiveness acceptability curves show the probability that the tailored lifestyle intervention is cost-effective in comparison with usual care for a range of different ceiling ratios thereby showing decision uncertainty [34].

The effect of the lifestyle intervention on long-term cardiovascular morbidity risk and costs as compared to usual care will be extrapolated over a time horizon of 10 years using a Markov model, if the study results indicate the intervention is effective. Health states that will be included in the model are BMI≤27, BMI>27, and death. Retention rates of the intervention will be based on the literature. Cardiovascular risk and costs for each health states, and transitions between health states will be

estimated based on the proposed study and existing literature. Probabilistic sensitivity analyses will be conducted to estimate uncertainty within the Markov model.

BUDGET IMPACT ANALYSIS (BIA)

General considerations BIA

A budget impact analysis (BIA) will be conducted from the perspective of health-care decision makers. The budget impact analysis will be performed based on the recommendations from Sullivan et al [35]. In the BIA, the size and characteristics of the study population will be estimated using available Dutch epidemiological data. In the budget impact analysis, the effectiveness of the treatments will be extrapolated over a period of 10 years based on the estimates obtained from the proposed study. Perspectives that will be considered are the societal and the government (Budget Kader Zorg) perspective. Different scenarios will be evaluated including the following: 1) the tailored lifestyle intervention is not implemented, i.e., all patients receive usual care, 2) the intervention is offered to the whole patient population, 3) the intervention is implemented over a 4 years period (25% of the patient population per year), and 4) the intervention is only offered to specific subgroups of the potential patient population. One-way sensitivity analyses will be performed in which the adoption rate of the tailored lifestyle intervention and the impact on long-term clinical outcomes will be varied.

Cost analysis BIA

The total number of patients eligible for the tailored lifestyle intervention will be estimated based on Dutch incidence and prevalence rates of SMI. Resource allocation is calculated by multiplying the number of eligible patients with the resource utilization rates obtained from the cost-effectiveness analysis. Different prices will be used to value resource use depending on the perspectives of the analysis: Dutch standard costs for the societal perspective and actual NZA tariffs for the government perspective. Both resource use and annual costs will be presented over a ten-year period for all perspectives. Aggregated and disaggregated (e.g. mental health care, medication, and laboratory tests) total costs per year will be presented for the different perspective and scenarios.

PROCESS EVALUATION

The study design also includes a process evaluation at the patient and FACT-team members level. Main objective is to examine the experiences and perceptions from patients and nurses with the lifestyle program. On patient level we will seek to understand participants' feeling toward, and responses to, the different elements of the lifestyle intervention, barriers and facilitators of dietary and exercise training, and to lifestyle change more generally. On the level of the FACT-team nurses we seek to understand satisfaction with the program, barriers and facilitators for application and implementation, assessment of intervention fidelity. Based on a purposive sampling strategy, semi-

structured interviews with 10-15 patients and the majority of nurses that performed the intervention will be conducted. All interviews will be audiotaped and transcribed.

ETHICAL CONSIDERATIONS

Regulation statement

This study is conducted in agreement with the declaration of Helsinki (2013), in accordance with the Dutch Medical Research Involving Human Subjects Act (WMO), and the Dutch Personal Data Protection Act (in Dutch: Wet Bescherming Persoonsgegevens). Patients will be able to contact an independent expert (prof. dr. Ralph Kupka, psychiatrist).

1.1 Recruitment and consent

Patients from the involved FACT-teams will be evaluated for suitability by Clinical Nurse Specialists, treating physicians and other healthcare staff from the according teams. The teams will select patients on BMI information and other inclusion/exclusion criteria found in electronic patient files. FACT-team members will ask if suitable patients are interested in joining the study and give permission to share their private (contact) information with the research team. The research team will telephone potential participants who gave persmission and ask to send information letters and informed consent formsIf needed the research team can inform patients by telephone contact. The study will make use of one information letter/informed consent for the FACT-team patients allocated in the intervention condition, and a different information letter/informed consent for FACT-team patients allocated in the control condition. In the information letter for patients in the control condition the information regarding the lifestyle intervention is left out. In the control group patients will only be asked if they are willing to perform measurements for a small reimbursement. Trained health care workers from the FACT teams and treating physicians or other can provide and discuss information with the patients and can answer any open questions if needed. Patients can always contact the researchers for more information as well, and will be provided with contact information of the research team in the information letter. Apart from that, patients will be able to contact an independent researcher for questions.

Patients can take up to five workdays to sign the informed consent. If patients are prepared to participate, informed consent procedure is followed, where the patient signs two informed consent forms or receives a copy. The patient information letter and informed consent form will be attached as a separate document. The informed consent will be obtained from each patient before the baseline assessment of the study begins. Patients can bring the informed consent form at their first baseline measurement. Assessors of the baseline measurements can supply additional information of the study if needed.

Benefits and risks assessment, group relatedness

Participants in the lifestyle intervention condition directly benefit from participating in the study by receiving guidance and information on food and food-related behaviours, physical activity and smoking cessation that might have an positive impact on eating habits, other lifestyle behaviours and well-being. As a result patients can lose weight on short term and might reduce cardiovascular risks. Risks associated with participation in this study are deemed minor. Possible extra risk associated with the intervention includes injury as a result of indoor or outdoor physical activity. However, as the primary physical activity is walking outdoors, this is considered a minor risk. Apart from that risks evolving venepuncture (such as minor bruising, hematoma, bleeding complications, fainting or infections) could occur. However patients involved in the study have annual laboratory check-ups. The laboratory assessments are measured at baseline and after 12 months, this study will make use of the patient's annual check-ups whenever possible in order to avoid extra visits to the laboratory.
The intervention itself can be challenging/exhausting for the participants, as it involves weekly group sessions of 2 hours in the first half year. However patients are clearly informed that they can stop with the study any time. Apart from that, healthcare staff from the FACT-teams can indicate if the burden becomes to high for a participant and advice the participant to stop the intervention. It is possible for nurses to assess this in the group sessions.
We think these risks are in proportion to the potential value of this study, which is looking at several lifestyle behaviours that might be effective in the prevention of cardiovascular disease in the SMI population. This could potentially be really important for mental health care in the future.

1.2 Incentives (if applicable)

Participants in the study will receive a small financial reimbursement for performing measurements during the study.

2. ADMINISTRATIVE ASPECTS, MONITORING AND PUBLICATION

2.1 Handling and storage of data and documents

All data handling is confidential and complies with the Dutch Personal Data Protection Act (in Dutch: De Wet Bescherming Persoonsgegevens, Wbp). Study data are collected and stored "coded" in locked cabinets or directly written in the electronic data management files. The "coded" forms (Case Report Forms) are identified by study number. Study numbers are composed of a 3 digit FACT-team specific code and a 4 digit patient specific code. Key to the study numbers is filed in a separate patient inclusion list in a locked cabinet in the study room or saved into a (shielded) electronic file. For mailing and planning purposes, there is one database with personal contact information about included patients, but with no clinical or study data. The METc will be informed of the start date of the study once the study commences.

Blood samples are not stored and saved for this study. Each laboratory follows their standard procedure as they do for patients annual check-ups. Therefore the samples are inmediately destroyed after assessment. This means nobody has access to this material during the research. The data in the database itself will be saved for 15 years.

Public disclosure and publication policy

The protocol of the study will be submitted for publication in an open access journal and the trial details will be registered in a public trial registry before start of patient inclusion. The results of the study (effectiveness and cost-effectiveness) will be publicly disclosed and submitted for publication in an open access scientific journal, independent of the outcome. Separate publications authored by appropriate study contributors might be pursued depending on the topic of the data.

3. REFERENCES

1. Laursen, T.M., M. Nordentoft, and P.B. Mortensen, *Excess early mortality in schizophrenia.* Annual review of clinical psychology, 2014. **10**: p. 425-448.
2. Walker, E.R., R.E. McGee, and B.G. Druss, *Mortality in mental disorders and global disease burden implications: a systematic review and meta-analysis.* JAMA psychiatry, 2015. **72**(4): p. 334-341.
3. Lawrence, D., K.J. Hancock, and S. Kisely, *The gap in life expectancy from preventable physical illness in psychiatric patients in Western Australia: retrospective analysis of population based registers.* 2013.
4. Lawrence, D.M., et al., *Death rate from ischaemic heart disease in Western Australian psychiatric patients 1980-1998.* The British journal of psychiatry, 2003. **182**(1): p. 31-36.
5. Saha, S., et al., *Are lifestyle interventions in primary care cost-effective?–An analysis based on a Markov model, differences-in-differences approach and the Swedish Björknäs study.* PloS one, 2013. **8**(11): p. e80672.
6. Tiihonen, J., et al., *11-year follow-up of mortality in patients with schizophrenia: a population-based cohort study (FIN11 study).* The Lancet, 2009. **374**(9690): p. 620-627.
7. De Hert, M., et al., *Cardiovascular disease and diabetes in people with severe mental illness position statement from the European Psychiatric Association (EPA), supported by the European Association for the Study of Diabetes (EASD) and the European Society of Cardiology (ESC).* European psychiatry, 2009. **24**(6): p. 412-424.
8. Cabassa, L.J., J.M. Ezell, and R. Lewis-Fernandez, *Lifestyle interventions for adults with serious mental illness: a systematic literature review.* Psychiatr Serv, 2010. **61**(8): p. 774-82.
9. Dunkley, A.J., et al., *Diabetes prevention in the real world: effectiveness of pragmatic lifestyle interventions for the prevention of type 2 diabetes and of the impact of adherence to guideline recommendations: a systematic review and meta-analysis.* Diabetes Care, 2014. **37**(4): p. 922-33.
10. Green, C.A., et al., *The STRIDE weight loss and lifestyle intervention for individuals taking antipsychotic medications: a randomized trial.* Am J Psychiatry, 2015. **172**(1): p. 71-81.
11. Schellenberg, E.S., et al., *Lifestyle interventions for patients with and at risk for type 2 diabetes: a systematic review and meta-analysis.* Ann Intern Med, 2013. **159**(8): p. 543-51.
12. Verhaeghe, N., et al., *Effectiveness and cost-effectiveness of lifestyle interventions on physical activity and eating habits in persons with severe mental disorders: a systematic review.* Int J Behav Nutr Phys Act, 2011. **8**: p. 28.
13. Ward, M.C., D.T. White, and B.G. Druss, *A meta-review of lifestyle interventions for cardiovascular risk factors in the general medical population: lessons for individuals with serious mental illness.* J Clin Psychiatry, 2015. **76**(4): p. e477-86.
14. Daumit, G.L., et al., *A behavioral weight-loss intervention in persons with serious mental illness.* N Engl J Med, 2013. **368**(17): p. 1594-602.
15. Bartels, S.J., et al., *Pragmatic replication trial of health promotion coaching for obesity in serious mental illness and maintenance of outcomes.* Am J Psychiatry, 2015. **172**(4): p. 344-52.
16. Delespaul, P.H. and E.P.A. de consensusgroep, *[Consensus regarding the definition of persons with severe mental illness and the number of such persons in the Netherlands].* Tijdschr Psychiatr, 2013. **55**(6): p. 427-38.
17. Appel, L.J., et al., *Effects of comprehensive lifestyle modification on blood pressure control: main results of the PREMIER clinical trial.* JAMA, 2003. **289**(16): p. 2083-93.
18. Casagrande, S.S., et al., *Randomized trial of achieving healthy lifestyles in psychiatric rehabilitation: the ACHIEVE trial.* BMC psychiatry, 2010. **10**(1): p. 1.

19. Appel, L.J., et al., *Effects of comprehensive lifestyle modification on blood pressure control: main results of the PREMIER clinical trial.* JAMA: Journal of the American Medical Association, 2003.
20. McGaghie, W.C., D.L. Watson, and R.G. Tharp, *Self-Directed Behavior: Self-Modification for Personal Adjustment*. 1974, JSTOR.
21. Prochaska, J.O. and W.F. Velicer, *The transtheoretical model of health behavior change.* American journal of health promotion, 1997. **12**(1): p. 38-48.
22. Burke, B.L., H. Arkowitz, and C. Dunn, *The efficacy of motivational interviewing and its adaptations: What we know so far.* Motivational interviewing: Preparing people for change, 2002. **2**: p. 217-250.
23. Rollnick, S., P. Mason, and C. Butler, *Health behavior change: a guide for practitioners*. 1999: Elsevier Health Sciences.
24. Rollnick, S. and W.R. Miller, *What is motivational interviewing?* Behavioural and cognitive Psychotherapy, 1995. **23**(04): p. 325-334.
25. V&VN, *multidisciplinaire richtlijn Leefstijl bij patiënten met een ernstige psychische aandoening.* Verpleegkundigen & Verzorgenden Nederland (V&VN), 2015. **2015**.
26. Ware Jr, J.E., M. Kosinski, and S.D. Keller, *A 12-Item Short-Form Health Survey: construction of scales and preliminary tests of reliability and validity.* Medical care, 1996. **34**(3): p. 220-233.
27. Farivar, S.S., H. Liu, and R.D. Hays, *Half standard deviation estimate of the minimally important difference in HRQOL scores?* Expert review of pharmacoeconomics & outcomes research, 2004. **4**(5): p. 515-523.
28. Rademakers, J., et al., *Measuring patient activation in the Netherlands: translation and validation of the American short form Patient Activation Measure (PAM13).* BMC Public Health, 2012. **12**(1): p. 1.
29. Bouwmans, C., et al., *Feasibility, reliability and validity of a questionnaire on healthcare consumption and productivity loss in patients with a psychiatric disorder (TiC-P).* BMC health services research, 2013. **13**(1): p. 217.
30. Zorginstituut and Nederland, *Richtlijnen voor het uitvoeren van economische evaluaties in de gezondheidszorg.* 2015.
31. Janssen, M., et al., *Measurement properties of the EQ-5D-5L compared to the EQ-5D-3L across eight patient groups: a multi-country study.* Quality of Life Research, 2013. **22**(7): p. 1717-1727.
32. Van Buuren, S., H.C. Boshuizen, and D.L. Knook, *Multiple imputation of missing blood pressure covariates in survival analysis.* Statistics in medicine, 1999. **18**(6): p. 681-694.
33. Stinnett, A.A. and J. Mullahy, *Net health benefits a new framework for the analysis of uncertainty in cost-effectiveness analysis.* Medical Decision Making, 1998. **18**(2): p. S68-S80.
34. Fenwick, E., B.J. O'Brien, and A. Briggs, *Cost‐effectiveness acceptability curves–facts, fallacies and frequently asked questions.* Health economics, 2004. **13**(5): p. 405-415.
35. Sullivan, S.D., et al., *Budget impact analysis—principles of good practice: report of the ISPOR 2012 Budget Impact Analysis Good Practice II Task Force.* Value in health, 2014. **17**(1): p. 5-14.
