# Supplementary material for: Effectiveness of a Lifestyle Intervention for People With a Severe Mental Illness in Dutch Outpatient Mental Health Care: A Randomized Clinical Trial
Source: JAMA Psychiatry. 2023 Jun 21;80(9):886–94. doi: 10.1001/jamapsychiatry.2023.1566 (PMC10285675; doi:10.1001/jamapsychiatry.2023.1566)
Supplement: Supplement 2. — eMethods. Overview Intervention Content eTable 1. Crude Analyses of Primary and Secondary Outcomes eTable 2. Adjusted Analyses of Primary Outcome per Covariate eTable 3. Crude Analyses of Clinically Relevant Weight Loss eTable 4. Adjusted Analyses of Clinically Relevant Weight Loss eTable 5. Total Characteristics of Complete Cases and Dropouts eTable 6. Characteristics of Complete Cases and Dropouts in the Intervention Group eTable 7. Characteristics of Complete Cases and Dropouts in the TAU Group eFigure. Unadjusted Mean Differences Over the 12-Month Period for Primary and Secondary Outcomes eReferences. [file jamapsychiatry-e231566-s002.pdf]

Supplemental Online Content

Walburg FS, van Meijel B, Hoekstra T, et al. Effectiveness of a lifestyle intervention for people with a severe mental illness in Dutch outpatient mental health care: a randomized clinical trial. *JAMA Psychiatry*. Published online June 21, 2023. doi:10.1001/jamapsychiatry.2023.1566

eMethods. Overview Intervention Content
eTable 1. Crude Analyses of Primary and Secondary Outcomes
eTable 2. Adjusted Analyses of Primary Outcome per Covariate
eTable 3. Crude Analyses of Clinically Relevant Weight Loss
eTable 4. Adjusted Analyses of Clinically Relevant Weight Loss
eTable 5. Total Characteristics of Complete Cases and Dropouts
eTable 6. Characteristics of Complete Cases and Dropouts in the Intervention Group
eTable 7. Characteristics of Complete Cases and Dropouts in the TAU Group
eFigure. Unadjusted Mean Differences Over the 12-Month Period for Primary and Secondary Outcomes
eReferences.

This supplementary material has been provided by the authors to give readers additional information about their work.

© 2023 Walburg FS et al. *JAMA Psychiatry*.

eMethods. Overview Intervention Content

SMILE study session content*

Weekly sessions (Initial phase)	Content of session
Session 1	Welcome and introduction to SMILE
Session 2	Portion sizes
Session 3	Energy balance and goal setting
Session 4	Breakfast, physical activity and regular eating
Session 5	Working on a healthy eating pattern
Session 6	Eating healthy on a budget
Session 7	Progress check
Session 8	Meal planning
Session 9	Your environment and social support
Session 10	Adverse effects of medication and weight gain
Session 11	Sleeping behavior and coping with stress
Session 12	Progress check and tackling negative thinking
Session 13	Eating consciously
Session 14	Over-eating and emotional eating
Session 15	Eating out
Session 16	Importance of physical activity
Session 17	Meal planning and portion sizes
Session 18	Progress check and problem solving
Session 19	Social support
Session 20	Pitfalls
Session 21	Stagnation of progression
Session 22	How to maintain weight loss
Session 23	Coping with changes in mental health status
Session 24	Celebrating successes

© 2023 Walburg FS et al. *JAMA Psychiatry*.

**Monthly sessions (Maintenance phase)**	
Session 25-30	Recap of former topics and free input

* Session content is based on the STRIDE intervention program materials (1)

© 2023 Walburg FS et al. *JAMA Psychiatry*.

eTable 1. Crude Analyses of Primary and Secondary Outcomes

eTable 1 Crude models at three, six and 12 months compared to baseline and overall effects on outcomes over the 12 month follow-up period.

Outcomes	Baseline - 3 months<br>B (95% CI)	Baseline - 6 months<br>B (95% CI)	Baseline - 12 months<br>B (95% CI)	Average difference [b]<br>B (95% CI)
Weight loss (kg)	-1.7 (-3.9 ; 0.5)	-1.9 (-4.1 ; 0.2)	-2.6 (-4.8 ; -0.4)	-2.1 (-4.0 ; -0.2)
BMI (kg/m2)		-0.7 (-1.4 ; 0,9)	-0.9 (-1.6 ; -0.1)	-0.7 (-1.3 ; -0.0)
Systolic blood pressure (mmHg)		1.8 (-1.9 ; 5.4)	1.6 (-2.2 ; 5.3)	1.6 (-1.1 ; 4.4)
Diastolic blood pressure (mmHg)		-1.0 (-3.4 ; 1.5)	0.5 (-2.0 ; 3.0)	0.1 (-1.8 ; 2.1)
Total cholesterol (mmol/L)			-0.3 (-0.6 ; -0.0)	
HDL cholesterol (mmol/L)			-0.0 (-0.1 ; 0.1)	
LDL cholesterol (mmol/L)			-0.3 (-0.6 ; -0.1)	
Triglycerides (mmol/L)			1.0 (0.91 – 1.18) [c]	
Fasting Glucose (mmol/L)			0.2 (-0.3 ; 0.7)	
Perceived physical health [d]		-0.1 (-0.7 ; 0.4)	0.1 (-0.4 ; 0.7)	0.1 (-0.5 ; 0.5)
Perceived mental health [d]		-0.0 (-0.5 ; 0.5)	-0.0 (-0.5 ; 0.5)	-0.0 (-0.4 ; 0.4)
Perceived healthy PA pattern [d]		0.2 (-0.4 ; 0.7)	0.3 (-0.2 ; 0.9)	0.3 (-0.2 ; 0.7)
Perceived healthy nutrition pattern [d]		0.04 (-0.4 ; 0.5)	0.1 (-0.4 ; 0.5)	0.0 (-0.4 ; 0.4)
Average sleep in last two weeks, h		-0.3 (-0.8 ; 0.2)	-0.5 (-1.0 ; -0.4)	-0.4 (-0.8 ; -0.0)

© 2023 Walburg FS et al. *JAMA Psychiatry*.

Satisfaction with weight [e]	0.8 (0.2 ; 1.5)	0.2 (-0.5 ; 0.8)	0.5 (-0.1 ; 1.1)
Satisfaction with PA behavior [e]	0.2 (-0.4 ; 0.8)	0.3 (-0.3 ; 0.9)	0.3 (-0.2 ; 0.7)
Satisfaction with dietary behavior [e]	0.0 (-0.5 ; 0.6)	0.1 (-0.5 ; 0.6)	0.1 (-0.4 ; 0.5)
Satisfaction with sleep pattern [e]	0.5 (-0.1 ; 1.2)	0.1 (-0.6 ; 0.8)	0.3 (-0.2 ; 0.9)
SF-12 Physical Component Summary [f]	0.5 (-2.2 ; 3.1)	0.2 (-2.5 ; 3.0)	0.3 (-1.9 ; 2.7)
SF-12 Mental Component Summary [f]	-2.0 (-5.4 ; 1.4)	-0.3 (-3.8 ; 3.1)	-1.2 (-4.1 ; 1.7)
PAM-13 (score) [g]	2.6 (-1.0 ; 6.1)	-0.9 (-4.5 ; 2.7)	0.9 (-2.3 ; 4.0)

Crude mixed model analyses

Abbreviations: BMI, Body Mass Index, PA, physical activity; h, hours; HDL, high-density lipoprotein; LDL, low-density lipoprotein; SF-12, Short-Form 12; PAM-13, Patient Activation Measure.

[a] Was corrected for baseline smoking status because smoking status was not measured at 3 months

[b] Average difference over time between the two groups

[c] Regression coefficients represented as ratios because of log-transformed Triglycerides

[d] Scored on a 0-10 numerical scale ranging from very unhealthy to very healthy based on the last 2 weeks

[e] Scored on a 0-10 numerical scale ranging from very dissatisfied to very satisfied

[f] Scores range from 0 to 100, with a higher score indicating better physical or mental health functioning

[g] Scores range from 0 to 100, with higher PAM scores indicating higher patient activation

© 2023 Walburg FS et al. *JAMA Psychiatry*.

eTable 2. Adjusted Analyses of Primary Outcome per Covariate

eTable 2. Adjusted models at 12 months for primary outcome per covariate compared to baseline

Outcomes	Baseline - 12months<br>B (95% CI)
Body weight change (kg) corrected for baseline and sex	-2.6 (-4.8 - -3.2)
Body weight change (kg) corrected for baseline and primary mental health diagnosis	-2.5 (-4.8 - -0.2)
Body weight change (kg) corrected for baseline and relationship status	-2.8 (-5.3 - -0.4)
Body weight change (kg) corrected for baseline and time-dependent smoking status	-2.9 (-5.5 - -0.4)
Body weight change (kg) corrected for baseline, sex, primary mental health diagnosis, relationship status and time-dependent smoking status (yes/no).	-3.3 (-6.2 - -0.4)

Adjusted mixed models corrected for baseline, sex, primary mental health diagnosis, relationship status and time-dependent smoking status (yes/no).

Abbreviations: CI, Confidence Interval; kg, kilograms

© 2023 Walburg FS et al. *JAMA Psychiatry*.

eTable 3. Crude Analyses of Clinically Relevant Weight Loss

eTable 3 Crude effects on ≥5% and ≥10% weight loss below baseline at six and 12 months

	**Baseline - 3 months**	**Baseline - 6 months**	**Baseline - 12 months**
	**OR (95%CI)**	**OR (95%CI)**	**OR (95%CI)**
≥5% weight loss below baseline	1.12 (0.46 ; 2.77)	2.08 (0.98 ; 4.34)	1.89 (0.89 ; 4.05)
≥10% weight loss below baseline	- [b]	4.56 (0.95 ; 21.78)	3.18 (1.06 ; 9.54)

Multivariable logistic regression analyses
Abbreviations: OR, Odds Ratio; CI, Confidence Interval

Results should be interpreted with caution because of small numbers and wide confidence intervals.

[b] As there were no cases with a ≥10% weight loss after 3 months in the control group, no logistic regression analysis was performed.

© 2023 Walburg FS et al. *JAMA Psychiatry*.

eTable 4. Adjusted Analyses of Clinically Relevant Weight Loss

eTable 4 Adjusted effects on ≥5% and ≥10% weight loss from baseline at 3, 6 and 12 months

			≥5% weight loss from baseline	≥10% weight loss from baseline
3 months	Intervention	N (%)	12 (13%)	2 [a] (2%)
	TAU	N (%)	10 (12%)	0 (0%)
		OR (95%CI)	1.3 (0.4 - 4.3)	- [b]
6 months	Intervention	N (%)	26 (28%)	9 (10%)
	TAU	N (%)	14 (16%)	2 [a] (2%)
		OR (95%CI)	2.2 (0.9 - 5.1)	3.1 (0.5 - 17.8)
12 months	Intervention	N (%)	22 (27%)	13 (16%)
	TAU	N (%)	14 (17%)	5 [a] (6%)
		OR (95%CI)	1.8 (0.8 - 4.3)	4.2 (1.0 - 16.9)

Multivariable logistic regression analyses adjusted for baseline weight, sex, primary mental health diagnosis, relationship (yes/no) and time independent smoking status (yes/no).

Abbreviations: TAU, Treatment as Usual; OR, Odds Ratio; CI, Confidence Interval;

Percentages (%) are calculated based on available data after follow-up.

[a] Results should be interpreted with caution because of small numbers and wide confidence intervals.

[b] As there were no cases with a ≥10% weight loss after 3 months in the control group, no logistic regression analysis was performed.

© 2023 Walburg FS et al. *JAMA Psychiatry*.

eTable 5. Total Characteristics of Complete Cases and Dropouts

eTable 5. Total characteristics of complete cases and drop-outs

Variable	Complete (n=169)	Drop-outs (n=55)
**Body weight, mean (SD)**	101.5 (19.1)	102.5 (17.6)
**Body mass index, mean (SD)**	34.4 (6.3)	36.4 (5.2)
**Systolic, mean (SD)**	132 (18.7)	131 (19.7)
**Age, mean (SD)**	48.6 (10.5)	44.4 (12.5)
**Sex female, n, (%)**	96 (57)	41 (75)
**Sex male, n, (%)**	73 (43)	14 (25)
**In a relationship, n (%)**	44 (29)	17 (39)
**Active Smokers, n (%)**	73 (43)	18 (36)

Complete is defined as: participants with available data after 12 months of primary outcome body weight
Drop-outs is defined as: participants with no available data after 12 months of primary outcome body weight

Percentages are calculated based on participants with available data of the corresponding covariate on baseline

© 2023 Walburg FS et al. *JAMA Psychiatry*.

eTable 6. Characteristics of Complete Cases and Dropouts in the Intervention Group

eTable 6. Characteristics of complete cases and drop-outs in the intervention group

**Variable**	**Complete** (n=83)	**Drop-outs** (n=43)
**Body weight, mean (SD)**	100.3 (20.4)	103.8 (17.8)
**Body mass index, mean (SD)**	34.8 (6.6)	36.7 (5.4)
**Systolic, mean (SD)**	133.6 (22)	134.6 (19.2)
**Age, mean (SD)**	48.7 (10.5)	45.7 (12.8)
**Sex female, n (%)**	54 (65.1)	34 (79.1)
**Sex male, n (%)**	29 (34.9)	9 (20.9)
**In a relationship, n (%)**	23 (29,9)	15 (42.9)
**Active Smokers, n (%)**	28 (33.7)	15 (37.5)

Complete is defined as: participants with available data after 12 months of primary outcome body weight in the intervention group
Drop-outs is defined as: participants with no available data after 12 months of primary outcome body weight in the intervention group

Percentages and means are calculated based on participants with available data of the corresponding covariate on baseline

© 2023 Walburg FS et al. *JAMA Psychiatry*.

eTable 7. Characteristics of Complete Cases and Dropouts in the TAU Group

eTable 7. Characteristics of complete and drop-outs in the TAU group

**Variable**	**Complete** (n=86)	**Drop-outs** (n=12)
**Body weight, mean (SD)**	102.6 (17.8)	96.9 (16.2)
**Body mass index, mean (SD)**	34.1 (6.0)	35.5 (4.2)
**Systolic, mean (SD)**	130.5 (15.1)	116.3 (14.3)
**Age, mean (SD)**	48.6 (10.5)	40.1 (10.4)
**Sex female, n (%)**	42 (48.8)	7 (58.3)
**Sex male, n (%)**	44 (51.2)	5 (41.7)
**In a relationship Yes (%)**	21 (27.3)	2 (22.2)
**Active Smokers Yes (%)**	45 (52.3)	3 (30.0)

Complete is defined as: participants with available data after 12 months of primary outcome body weight in the TAU group
Drop-outs is defined as: participants with no available data after 12 months of primary outcome body weight in the TAU group

Percentages are calculated based on participants with available data of the corresponding covariate on baseline

© 2023 Walburg FS et al. *JAMA Psychiatry*.

eFigure. Unadjusted Mean Differences Over the 12-Month Period for Primary and Secondary Outcomes

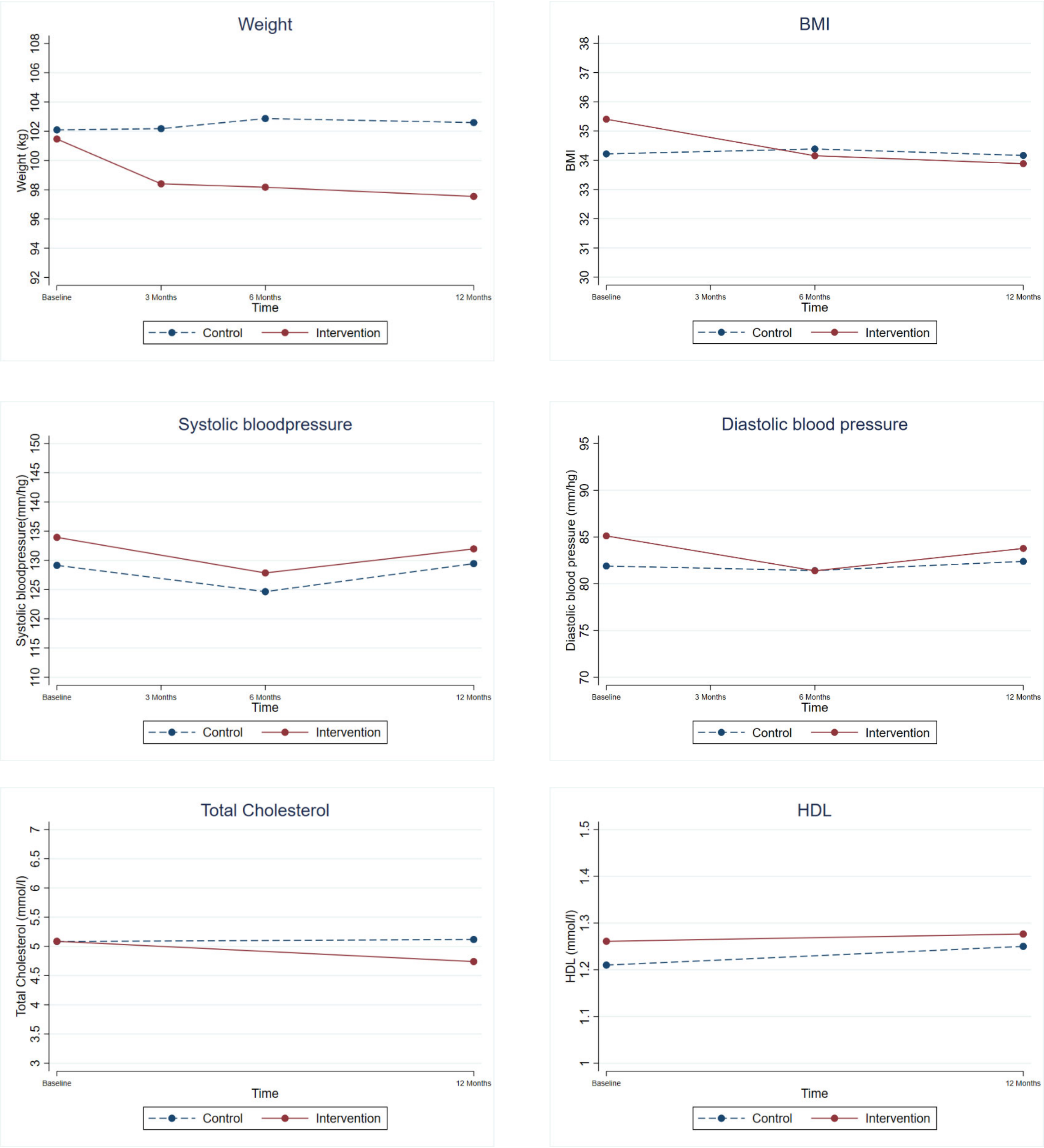

© 2023 Walburg FS et al. *JAMA Psychiatry*.

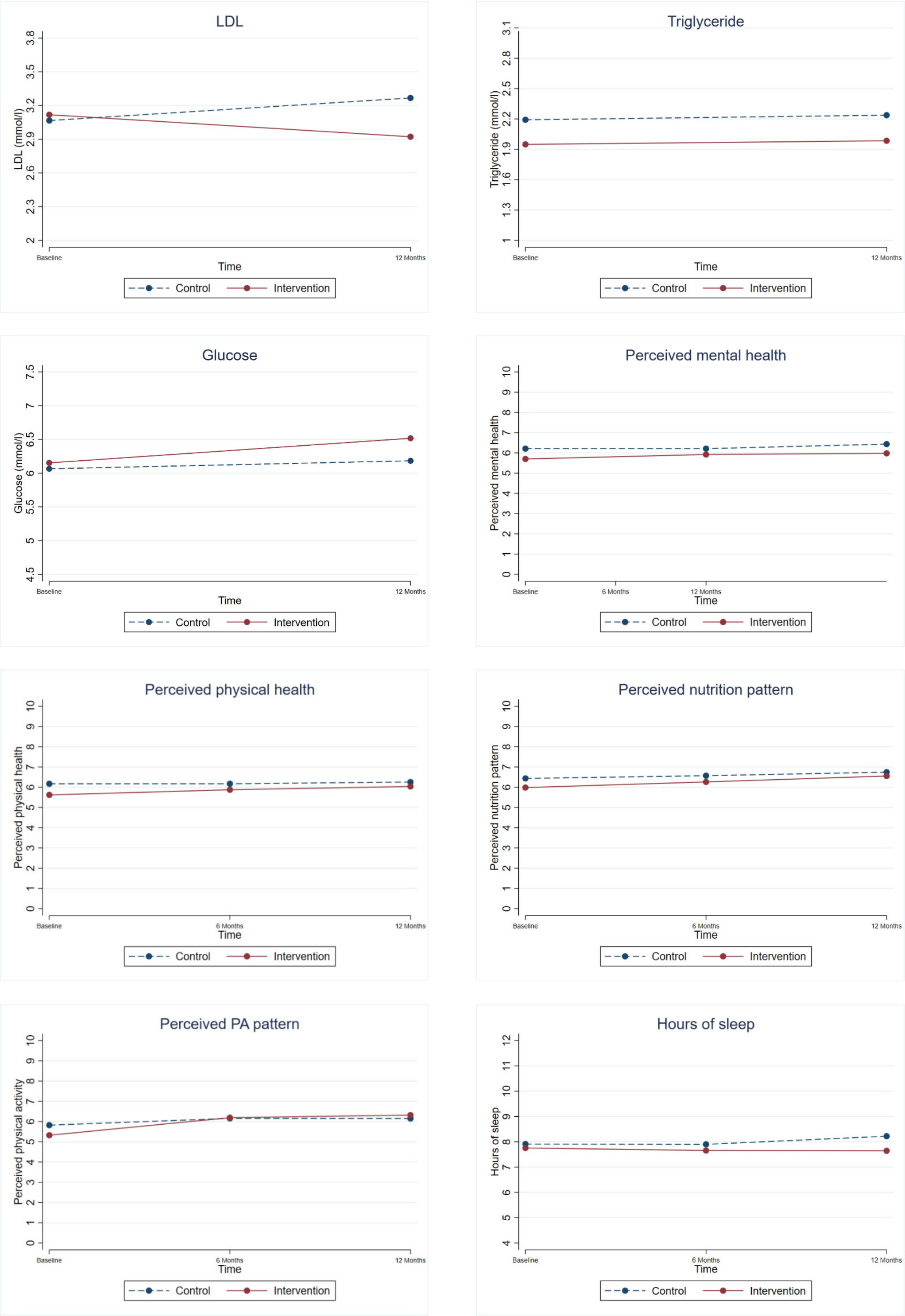

© 2023 Walburg FS et al. *JAMA Psychiatry*.

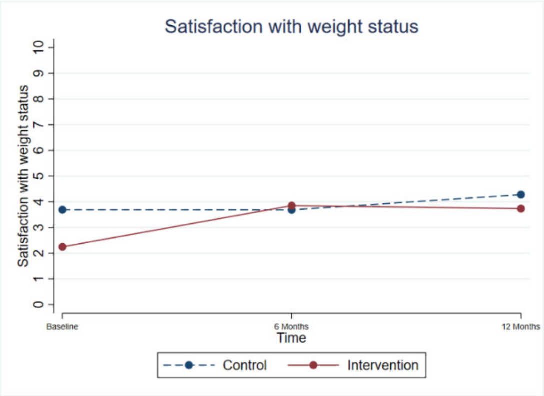

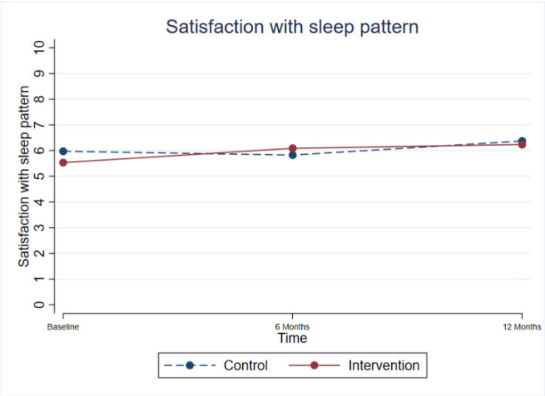

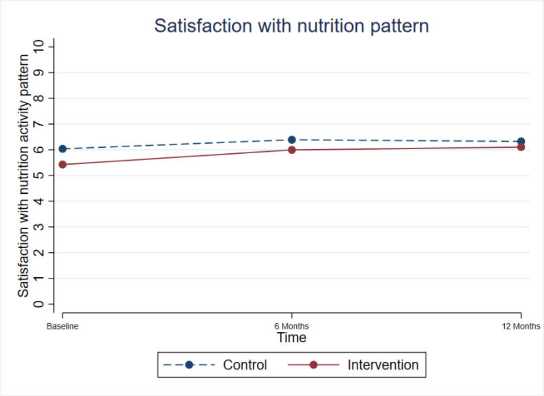

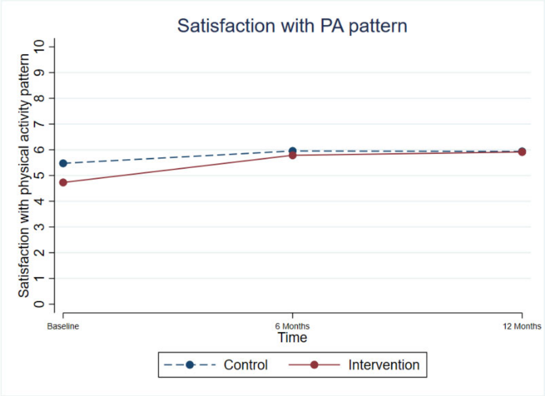

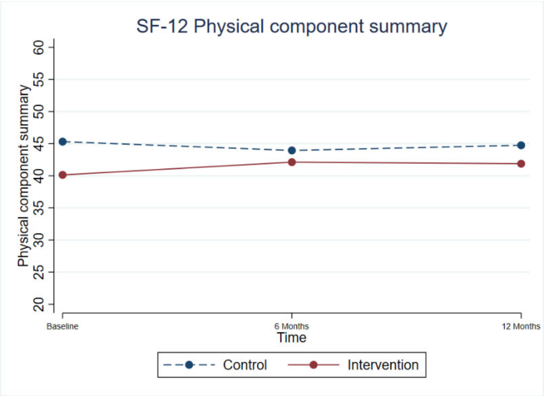

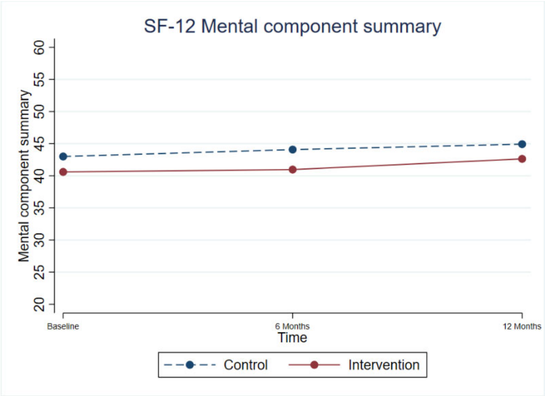

© 2023 Walburg FS et al. *JAMA Psychiatry*.

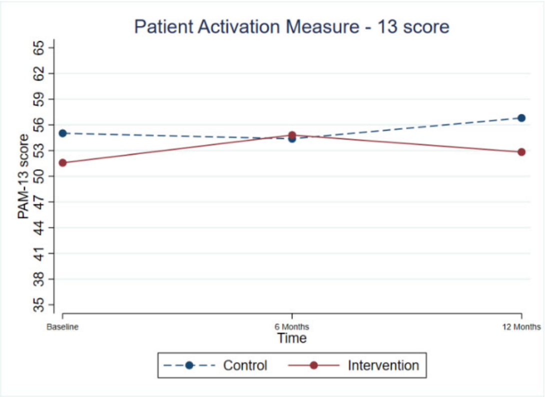

© 2023 Walburg FS et al. *JAMA Psychiatry*.

eReferences.

1. Yarborough BJH YM, Tehrani K, Funk KL, Stevens VJ, Green CA. Facilitator Guide for the STRIDE Program: A 30-Session Weight Loss and Weight Maintenance Program for People who live with Mental Illness. Portland (OR): Kaiser Permanente Center for Health Research; 2013. https://research.kpchr.org/Research/Research-Areas/Mental-Health/STRIDE
