# Supplementary material for: Effectiveness of a Lifestyle Intervention for People With a Severe Mental Illness in Dutch Outpatient Mental Health Care: A Randomized Clinical Trial
Source: JAMA Psychiatry. 2023 Jun 21;80(9):886–94. doi: 10.1001/jamapsychiatry.2023.1566 (PMC10285675; doi:10.1001/jamapsychiatry.2023.1566)
Supplement: Supplement 3. — Data Sharing Statement [file jamapsychiatry-e231566-s003.pdf]

Data Sharing Statement

Walburg. Effectiveness of a Lifestyle Intervention for People With a Severe Mental Illness in Dutch Outpatient Mental Health Care. *JAMA Psychiatry*. Published June 21, 2023.
doi:10.1001/jamapsychiatry.2023.1566

Data
Data available: Yes
Data types: Deidentified participant data
How to access data: florine.walburg@vu.nl
When available: With publication

Supporting Documents
Document types: None

Additional Information
Who can access the data: Researchers whose proposed use of the data has been approved
Types of analyses: The datasets will be available on well-substantiated request
Mechanisms of data availability: with investigator support, after approval of a proposal and signed data access agreement
